# HIST3H2A promotes the progression of prostate cancer through inhibiting cell necroptosis

**DOI:** 10.1186/s12885-024-12308-4

**Published:** 2024-04-29

**Authors:** Lihong Yang, Yong Ruan, Houqiang Xu

**Affiliations:** 1 Key Laboratory of Animal Genetics, Breeding and Reproduction in the Plateau Mountainous Region, Ministry of Education, College of Life Sciences, Guizhou University, Guiyang, 550025 China; 2 College of Animal Science, Guizhou University, Guiyang, 550025 China

**Keywords:** Prostate cancer, HIST3H2A, Progression, Necroptosis

## Abstract

In recent years, there has been an increase in the incidence and mortality rates of prostate cancer (PCa). However, the specific molecular mechanisms underlying its occurrence and development remain unclear, necessitating the identification of new therapeutic targets. Through bioinformatics analysis, we discovered a previously unstudied differential gene called HIST3H2A in prostate cancer. Our study revealed that HIST3H2A is highly expressed in PCa tissues, as confirmed by analysis of both the GEO and UALCAN databases. Further analysis using the KEGG database demonstrated that HIST3H2A regulates the pathway of programmed necroptosis in cells. Additionally, we observed significant up-regulation of HIST3H2A in PCa tissues and cell lines. HIST3H2A was found to regulate cell proliferation, migration, invasion, and the epithelial-mesenchymal transition (EMT) process in tumors. Notably, HIST3H2A’s role in regulating programmed necroptosis in prostate cancer cells differs from its role in apoptosis. In vitro and in vivo experiments collectively support the key role of HIST3H2A in promoting the development of prostate cancer, highlighting its potential as a therapeutic target for patients with PCa.

**Supplementary Information:**

The online version contains supplementary material available at 10.1186/s12885-024-12308-4.

## Introduction

Prostate cancer (PCa) is the second most common cancer in men worldwide, with increasing incidence and mortality rates [1–3]. Despite advancements in diagnosis and treatment, the molecular mechanisms underlying prostate cancer development remain incompletely understood. Therefore, there is a critical need to identify new potential biomarkers and uncover unknown pathogenic factors. Mammalian cells undergo different forms of cell death, such as apoptosis, necroptosis, and pyroptosis, under various stress conditions [4–7]. Dysregulation of cell death processes can contribute to various human diseases, including cancer, neurodegeneration, and infectious diseases [8–11]. In the context of cell necroptosis, the protein kinase Receptor-interacting protein 3 (RIP3) plays a crucial role [12–14]. RIP3 is a serine/threonine kinase that consists of a homologous N-terminal kinase domain and a unique C-terminal domain [15–17]. Upon activation, RIP3 recruits and phosphorylates the lineage kinase domain-like protein (MLKL), leading to its oligomerization and localization to the plasma membrane. Ultimately, this results in cell membrane rupture and cell death [15, 18–20].

Nucleosomes, composed of histones H2A, H2B, H3, and H4, are the fundamental units of chromatin fibers in eukaryotic cells [21–23]. Each nucleosome consists of two units of H3 and H4, forming a tetramer surrounded by two dimers of H2A-H2B [24–26]. Variants of H2A, H2B, and H3 have been identified in eukaryotes, and certain variants of H2A have been linked to cancer [27, 28]. For instance, H2A.z is believed to have pro-tumor effects [29–31], while macroH2A and H2A.x are considered tumor inhibitors [32]. Deletion or mutation of the H2A.X gene can lead to severe DNA damage and has been associated with the development of various cancers, including breast cancer, head and neck squamous cell carcinoma, neuroblastoma, and hematopoietic malignancies [33–39]. Although the involvement of H2A family protein variants, such as H2A.Z-1 and H2A.Z-2, in prostate cancer has been established, the HIST3H2A underlying mechanism remains unclear.

In this study, we investigated the significance of HIST3H2A expression in prostate cancer. Our findings demonstrate that HIST3H2A expression may influence the occurrence and progression of prostate cancer. Specifically, HIST3H2A is significantly up-regulated in both human prostate cancer tissues and prostate cancer cells. Overexpression of HIST3H2A promotes cell proliferation, migration, and invasion, whereas interference with HIST3H2A inhibits the proliferation of prostate cancer cells and significantly reduces their migration and invasion capabilities. Notably, this process is mediated through programmed necroptosis rather than apoptosis. Collectively, our study suggests that HIST3H2A plays a crucial role in inducing cell death and could serve as a potential therapeutic target for prostate cancer.

## Methods and materials

### Data sources

The PCa mRNA datasets GSE 69,223, GSE 32,571, GSE 6919, and GSE 46,602 were obtained from the GEO database (https://www.ncbi.nlm.nih.gov/geo/). Subsequently, the UALCAN database (https://ualcan.path.uab.edu) was utilized to analyze the expression differences between cancerous and non-cancerous tissues. Additionally, the KEGG database was employed to identify the pathways regulated by HIST3H2A.

### Tissue and cell lines

Tissue chips were purchased from Servicebio Biotechnology Co., LTD (Wuhan, China). Human prostate cancer cell lines (PC3, 22RV1) and human normal prostate epithelial RWPE-1 cells were obtained from Zhongqiao Xinzhou Biotechnology (Shanghai, China).

### Cell culture and transfection

PC3 cells were cultured in DMEM-F12 medium (Gibco, NY, USA) supplemented with 10% fetal bovine serum (VivaCell, China) and 1% streptomycin-penicillin (Gibco, NY, USA). 22RV1 cells were cultured in RPMI-1640 medium (Gibco, NY, USA) supplemented with 10% fetal bovine serum (VivaCell, China) and 1% streptomycin-penicillin (Gibco, NY, USA). RWPE-1 cells were sustained in a specialized medium for RWPE-1 cells (ZQ-1303, ZQXZ Bio, Shanghai, China). All cells were cultured at 37 °C in a CO_2_ incubator with 5% CO_2_. pcDNA3.1-HIST3H2A (oe-HIST3H2A) and LV-HIST3H2A vectors were obtained from Tsingke (Chongqing, China), and corresponding negative controls (NC) were also obtained. Cells were transfected with the specific vector using FuGENE HD (Promega, Madison, WI, USA) reagents following the instructions for use, and then used for further research.

### Real-time quantitative PCR (qRTPCR)

Total RNA was extracted from PC3, 22RV1, and RWPE-1 cells using the Trizol method (Invitrogen, Carlsbad, CA, USA) following the manufacturer’s instructions. RNA quality was assessed using ultraviolet spectrophotometry (Invitrogen, Carlsbad, CA, USA). For RNA reverse transcription, the RevertAid First Strand cDNA Synthesis kit from Invitrogen was employed. The qRT-PCR experiment was conducted using the CFX-96 Real-Time PCR system and Bio-Rad Laboratories SYBR Green Mix, with specific thermal cycle conditions set. The initial predenaturation step was performed at 95 °C for approximately 3 min, followed by 39 cycles of 30 s at 95 °C, 5 s at 57 °C, and 30 s at 72 °C. Three independent replicates were collected in this study, and mRNA expression levels were measured using GAPDH as an internal control. Data were analyzed using the 2^−ΔΔCq^ method [40]. The primers used for real-time PCR are listed in Table 1.

**Table 1:** The primers used for real-time PCR

Primer	Sequence	Product size (bp)
GAPDH-F	TGCAACCGGGAAGGAAATGA	148
GAPDH-R	GCATCACCCGGAGGAGAAAT	
HIST3H2A-F	GTCTCGCTTTTCGGTTGCC	74
HIST3H2A-R	CTTGCCACCCTGCTTACCA	

### Western blot

Proteins were extracted from cells using the radioimmunoprecipitation assay (RIPA) lysis buffer (Solarbio, China). The concentrations were determined using the bicinchoninic acid (BCA) protein assay (Solarbio, China). The extracted proteins were then electrophoresed on SDS-polyacrylamide gels and transferred to PVDF membranes (Millipore, Carrigtwohill, Ireland). After sealing with skim milk, the membranes were incubated overnight at 4 °C with the primary antibody. Subsequently, the membranes were washed and incubated with the secondary antibody at room temperature for 2 h. The antibodies used in this study were as follows: anti-HIST3H2A (1:1000 dilution; Proteintech, USA), anti-Bax (1:5000 dilution; Proteintech, USA), anti-MMP-9 (1:1000 dilution; Proteintech, USA), anti-MMP-2 (1:1000 dilution; Proteintech, USA), anti-E-cadherin (1:5000 dilution; Proteintech, USA), anti-N-cadherin (1:1000 dilution; Proteintech, USA), anti-PCNA (1:3000 dilution; Proteintech, USA), anti-Vimentin (1:5000 dilution; Proteintech, USA), anti-Caspase 9 (1:1000 dilution; Proteintech, USA), anti-CDK6 (1:5000 dilution; Proteintech, USA), anti-Cyclin D1 (1:5000 dilution; Proteintech, USA), anti-Bcl-2 (1:2000 dilution; Proteintech, USA), anti-Cyclin E1 (1:1000 dilution; Proteintech, USA), anti-CDK2 (1:1000 dilution; Proteintech, USA), GAPDH (1:1000 dilution; Proteintech, USA), and goat-anti-rabbit, goat-anti-mouse secondary antibody (1:10000 dilution; Proteintech, USA), anti-RIP3 (1:2000 dilution; Proteintech, USA), anti-p-RIP3 (1:2000 dilution; Affinity, USA), anti-MLKL (1:5000 dilution; Proteintech, USA), and anti-p-MLKL (1:1000 dilution; Affinity, USA). The results were quantified and the images were processed using Image J software.

### Immunohistochemistry

Immunohistochemistry (IHC) was employed to section mouse tumor tissue as described in a previous study [41]. The primary antibody used for IHC was the same as the one used for the western blot analysis. Tissue sections were incubated overnight at 4 °C with the primary antibody, followed by incubation with the secondary antibody. The DAB complex was utilized as a chromogen to visualize the target antigen. Nuclei were counterstained with hematoxylin.

### EdU assay

The impact of HIST3H2A on the proliferation of PC3 and 22RV1 cells was assessed using the EdU (APExBIO, USA) methods. In the EdU detection, the cells were transfected with either vector or shRNA for 48 h before being seeded in 24-well plates at a density of 2 × 10^5^ cells per well. The cells were then incubated with 20 mM EdU for 2 h. After fixing with 3.7% formaldehyde for 15 min and permeabilizing with 0.25% Triton X-100 for 15 min at room temperature, the cells were washed 3 times with PBS and incubated with Click buffer for 30 min at room temperature. The uptake rate of EdU was determined by calculating the ratio of the total number of EdU-positive cells (red) to the total number of blue cells after Hoechst 33,258 staining.

### Cell migration and invasion assays

After transfection, 1.5 × 10^5^ PC3 and 22RV1 cells were cultured in 180 µL of complete culture medium and inoculated into the upper chambers of a transwell (pore size 8 mm, Costar). Matrigel was added to the upper chambers for invasion experiments, but not for migration experiments. A chemo-attractant consisting of 600 µL complete growth medium and 15% fetal bovine serum was added to the bottom well of each chamber. The cells were then incubated for 24 h at 37 °C and 5% CO2 for migration and invasion assays. After incubation, the cells from the upper chamber were scraped off with a cotton swab. The invasive cells on the other side were fixed with 3.7% paraformaldehyde, stained with Giemsa, and photographed using a microscope (Nikon, Japan).

### Xenograft tumor model

The animal experiment was conducted in accordance with the guidelines set by the Committee for Animal Care and Use of Guizhou University according to the Basel Declaration. To establish the mouse tumor model, 5-week-old BALB/c nude mice (Tengxin Biotechnology, Chongqing, China) were randomly divided into two groups (*n* = 7 per group): one group received cells with Lentiviral shRNA control vector (HIST3H2A-NC) and the other group received cells with Lentiviral shRNA vector (HIST3H2A-shRNA). The cells were injected subcutaneously into the nude mice at a concentration of 4 × 10^6^ cells in 0.2 mL of PBS per mouse. At the end of the 4-week period, the mice were euthanized using an overdose of pentobarbital (250 mg/kg intraperitoneal injection). Once it was confirmed that the mice had passed away as a result of respiratory and cardiac arrest, the tumor tissues were collected and subjected to immunohistochemistry (IHC) staining.

### Statistical analysis

The statistical data was obtained from triple repetitions using GraphPad Prism 9 (GraphPad, La Jolla, CA, USA) and is presented as mean ± SD. To analyze the statistical significance differences between the two datasets, a two-tailed Student’s t-test was performed. A *p*-value of less than 0.05 was considered statistically significant.

## Results

### Screening and expression analysis of HIST3H2A

**Fig. 1:**
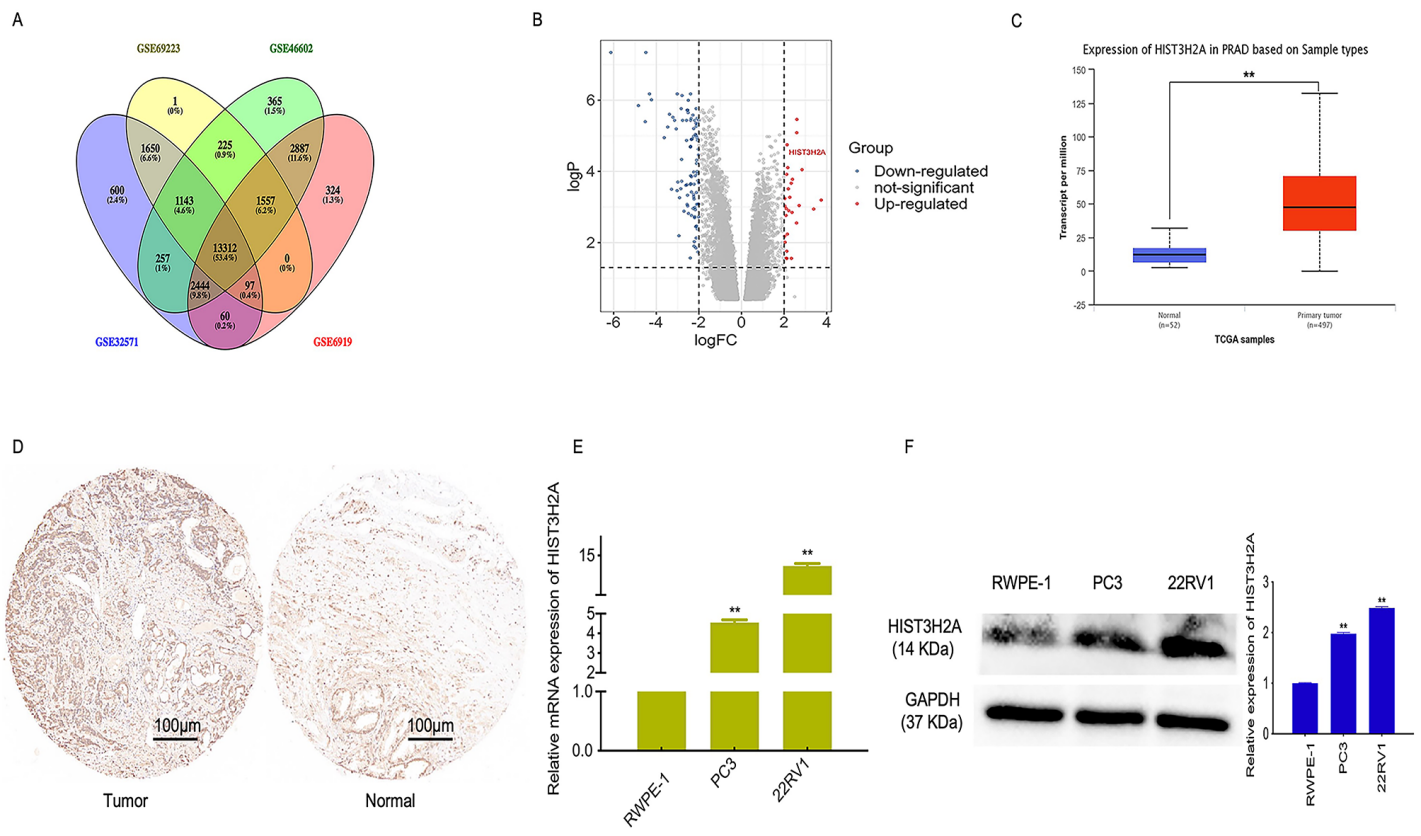
Screening and expression analysis of HIST3H2A. **A** Differential gene screening. **B** Fold change of differential gene. **C** Analysis of expression difference between cancer and paracancer in UALCAN database. **D** HIST3H2A expression between PCa tumor tissue and adjacent non-tumor tissue by tissue chip IHC. **E** HIST3H2A expression level detected in prostate cancer cell lines (PC3 and 22RV1) and normal prostate RWPE-1 cells by qRT-PCR. **F** HIST3H2A expression level detected in prostate cancer cell lines (PC3 and 22RV1) and normal prostate RWPE-1 cells by western blot. Scale bars 100 μm. *p < 0.05, **p < 0.01 by Student’s t-test. Error bars indicate SD

To identify potential molecular targets for prostate cancer, we conducted bioinformatics analyses using four databases: GSE 69,223, GSE 32,571, GSE 6919, and GSE 46,602. Our analysis revealed that there were 13,312 genes common to all four databases (Fig. 1A). Among these genes, we found that HIST3H2A was highly expressed in prostate cancer and had not been previously studied in this context (Fig. 1B). Further analysis using the UALCAN database confirmed that HIST3H2A expression in prostate cancer was significantly higher than in adjacent prostate cancer tissues (Fig. 1C). To validate these findings, we performed additional experiments using tissue microarray IHC (Tumor, 59 point and Normal, 27 point), qRT-PCR, and western blot. Consistently, our results showed that HIST3H2A expression was significantly higher in prostate cancer tissues compared to adjacent prostate cancer tissues (Fig. 1D). Moreover, both qRT-PCR and western blot analyses demonstrated that HIST3H2A expression was significantly higher in PC3 and 22RV1 cells, two prostate cancer cell lines, compared to normal prostate RWPE-1 cells (Fig. 1E and F).

### HIST3H2A regulates cells proliferation

**Fig. 2:**
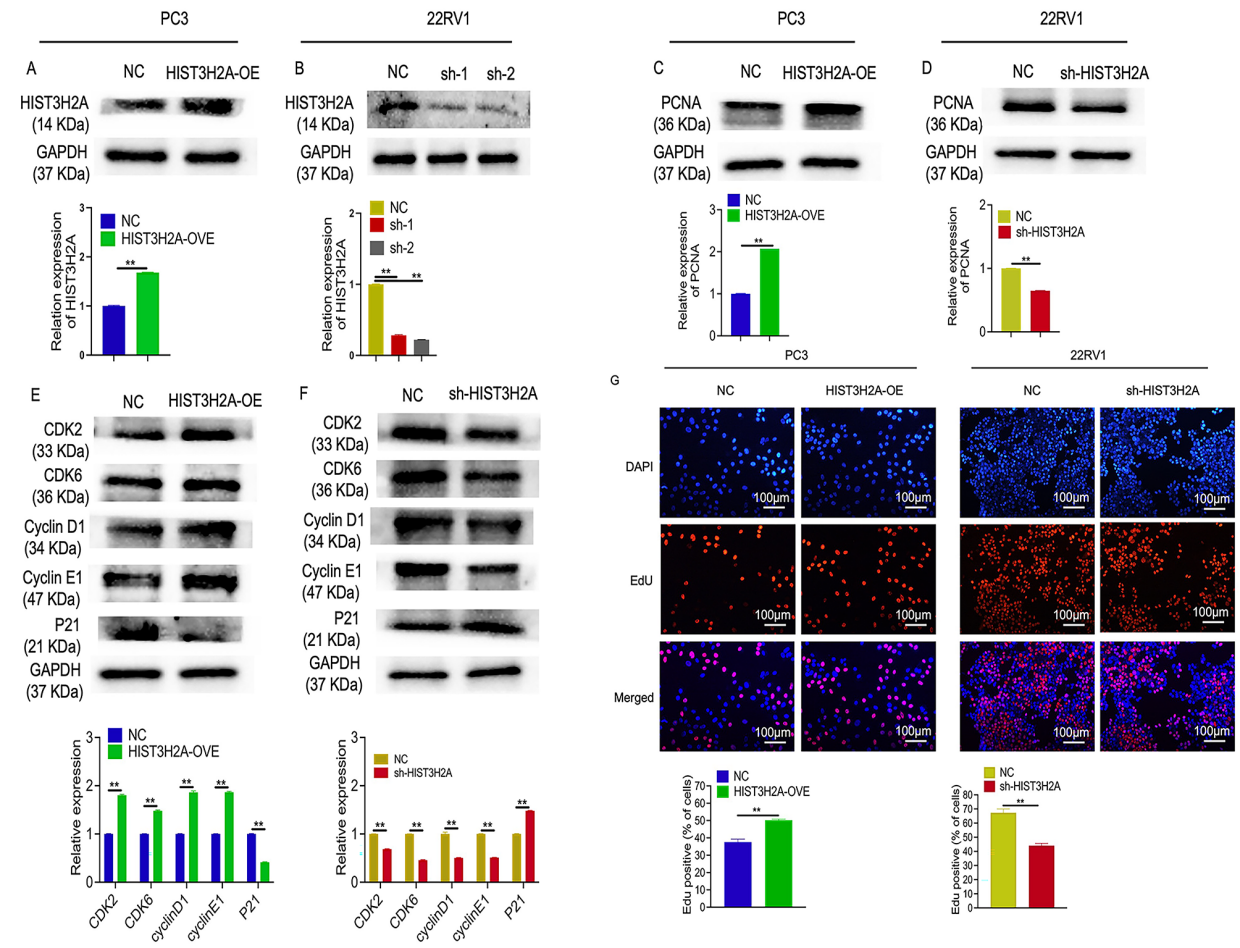
HIST3H2A regulates Cells proliferation. **A** HIST3H2A overexpression efficiency detection by western blot. **B** HIST3H2A interference efficiency detection by western blot. **C** The effect of HIST3H2A overexpression on the proliferation-related gene by western blot. **D** The effect of HIST3H2A interference on the proliferation-related gene by western blot. **E** The effect of HIST3H2A overexpression on the cycle-related gene by western blot. **F** The effect of HIST3H2A interference on the cycle-related gene by western blot. **G** The effect of HIST3H2A overexpression or knockdown on the proliferation of PC3 and 22RV1 cells were assessed by EdU. Scale bars 100 μm. *p < 0.05, **p < 0.01 by Student’s t-test

To investigate the role of HIST3H2A in cell proliferation, we conducted several assays including western blot, 5-ethynyl-20-deoxyuridine (EdU) incorporation. The western blot analysis revealed that HIST3H2A was overexpressed in PC3 cells (Fig. 2A), while successful interference with HIST3H2A was achieved in 22RV1 cells (Fig. 2B). Overexpression of HIST3H2A in PC3 cells resulted in increased expression of proliferation-related proteins such as PCNA, CDK2, CDK6, cyclinD1, cyclinE1, and decreased expression P21 (Fig. 2C and E). Conversely, interference with HIST3H2A in 22RV1 cells led to the opposite result (Fig. 2D and F). Similar findings were observed through EdU incorporation assay (Fig. 2G).

### HIST3H2A regulates EMT process

**Fig. 3:**
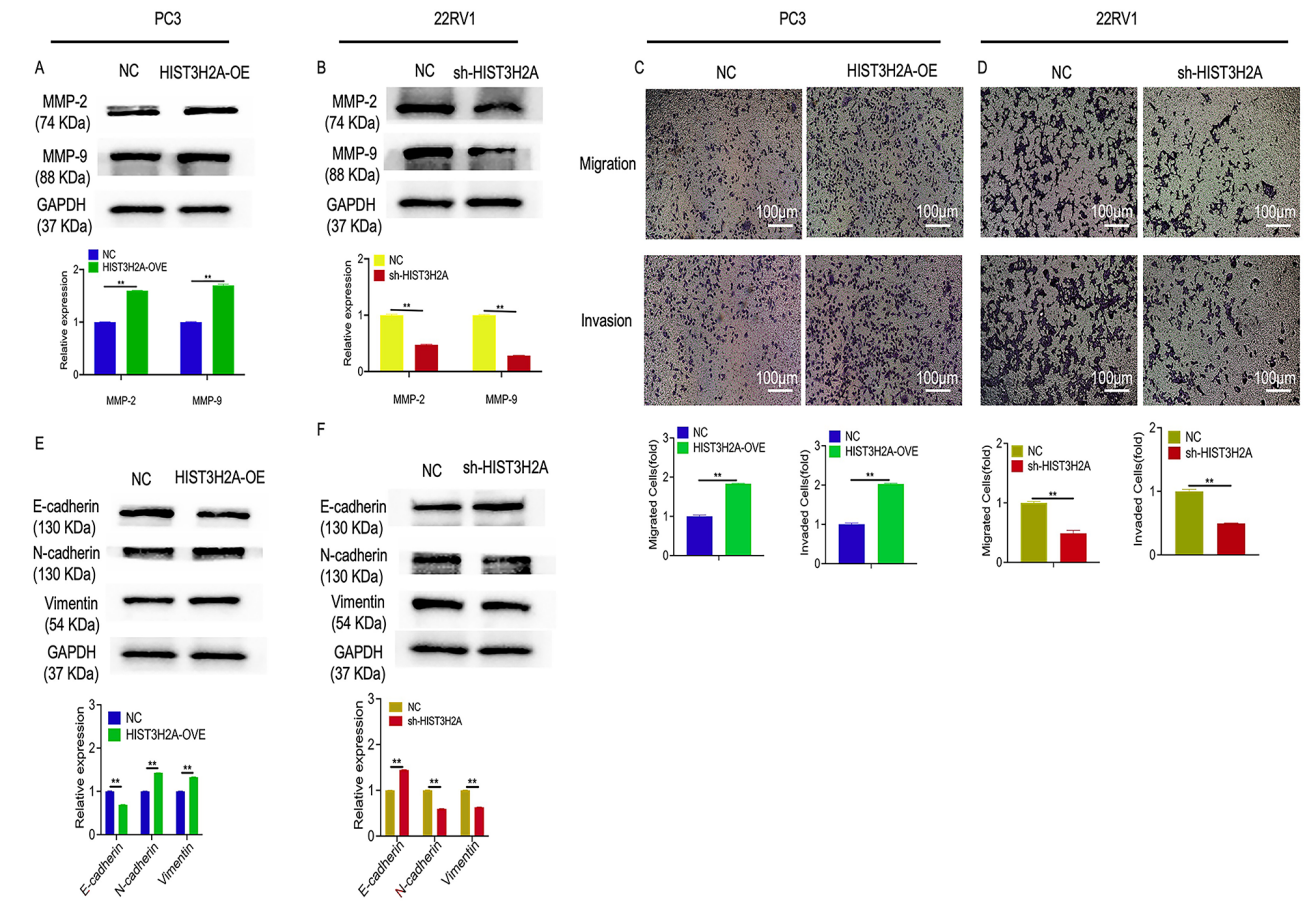
HIST3H2A regulates EMT process. **A** The effect of HIST3H2A overexpression on the tumor invasion and metastasis genes by western blot. **B** The effect of HIST3H2A interference on the tumor invasion and metastasis genes by western blot. **C** The effect of HIST3H2A overexpression on the migration and invasion by transwell assay. **D** The effect of HIST3H2A interference on the migration and invasion by transwell assay. **E** The effect of HIST3H2A overexpression on the EMT-related gene by western blot. **F** The effect of HIST3H2A interference on the EMT-related gene by western blot. Scale bars 100 μm

When tumor cells invade and metastasize, they tend to lose polarity and epithelial characteristics, such as changes in the expression of proteins like E-cadherin, N-cadherin, and Vimentin, and acquire mesenchymal phenotypes similar to the enhancement of MMP expression. This phenomenon is known as the epithelial-to-mesenchymal transition (EMT). In this study, western blot and transwell tests were conducted to detect cell invasion, migration, and metastasis. The western blot results showed that overexpression of HIST3H2A in PC3 cells significantly increased the expression levels of invasion-related genes MMP-2 and MMP-9, decreased the expression levels of E-cadherin, and increased the expressions of N-cadherin and Vimentin (Fig. 3A and E). On the other hand, interference of HIST3H2A in 22RV1 cells led to a significant decrease in the expression levels of invasion-related genes MMP-2 and MMP-9, an increase in the expression levels of E-cadherin, and a decrease in the expression levels of N-cadherin and Vimentin (Fig. 3B and F). The Transwell assay also demonstrated that overexpression of HIST3H2A promoted the invasion and migration of PC3 cells (Fig. 3C), while interference with HIST3H2A inhibited the invasion and migration of 22RV1 cells (Fig. 3D).

### HIST3H2A regulates cells proliferation through necroptosis but not through apoptosis

**Fig. 4:**
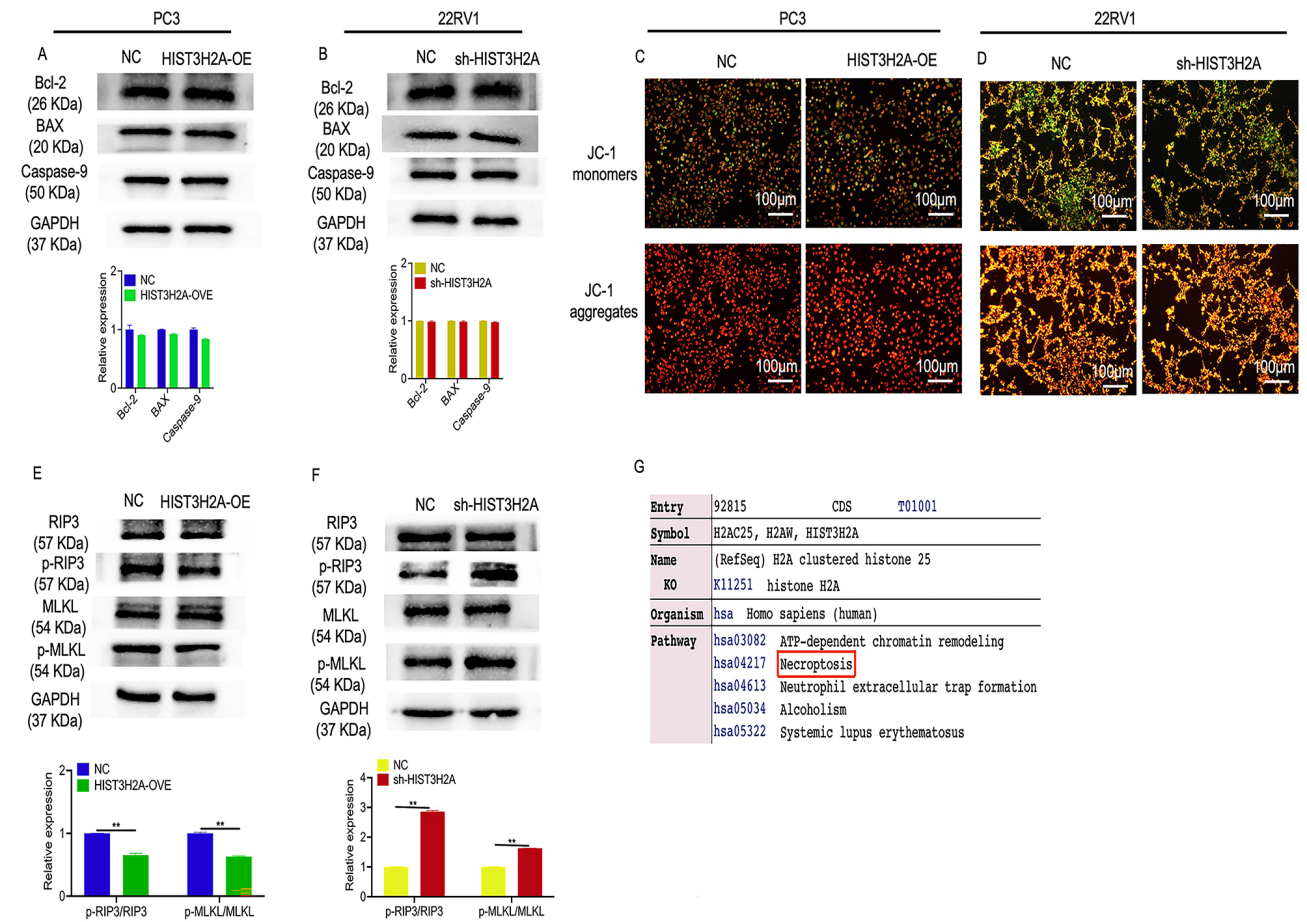
HIST3H2A regulates cells proliferation through necroptosis but not through apoptosis. **A** The effect of HIST3H2A overexpression on the apoptosis-related gene by western blot. **B** The effect of HIST3H2A interference on the apoptosis-related gene by western blot. **C** The effect of HIST3H2A overexpression on the apoptosis by JC-1 assay. **D** The effect of HIST3H2A interference on the apoptosis by JC-1 assay. **E** The effect of HIST3H2A overexpression on the necroptosis-related gene by western blot. **F** The effect of HIST3H2A interference on the necroptosis-related gene by western blot. **G** KEGG database showed that HIST3H2A was involved in necroptosis regulation. Scale bars 100 μm

To investigate the role of HIST3H2A in regulating the proliferation of prostate cancer cells, we examined the expression of apoptosis-related proteins Caspase 9, Bax, and Bcl-2. Western blot analysis revealed that overexpression or interference of HIST3H2A had no significant effect on the levels of these proteins in PC3 and 22RV1 cells (Fig. 4A and B). Additionally, the results of JC-1 staining showed that HIST3H2A interference or overexpression did not impact cell apoptosis (Fig. 4C and D). These findings suggest that HIST3H2A does not influence prostate cancer cell proliferation through apoptosis regulation. However, according to the KEGG database, HIST3H2A may be involved in the regulation of necroptosis (Fig. 4G). To further explore this, we performed western blot analysis to assess necroptosis-related proteins. We observed that the expression of RIP3, p-RIP3, MLKL, and p-MLKL decreased upon HIST3H2A overexpression in PC3 cells (Fig. 4E), while it increased after HIST3H2A interference in 22RV1 cells (Fig. 4A and F). Overall, our results suggest that HIST3H2A regulates cell proliferation through necroptosis rather than apoptosis.

### HIST3H2A regulates tumor growth in vivo

**Fig. 5:**
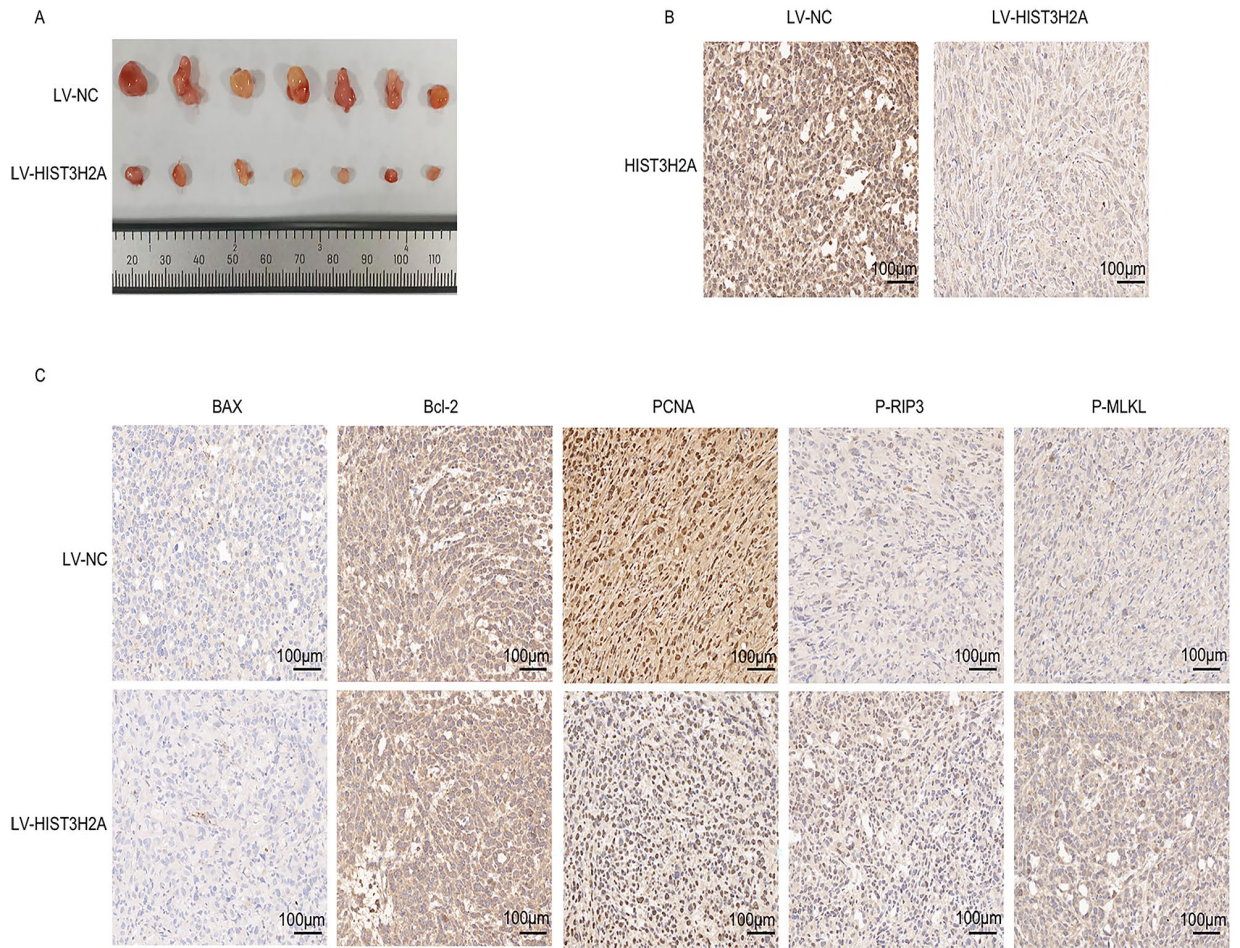
HIST3H2A regulates cells proliferation through necroptosis but not through apoptosis in vivo. **A** Images of a given set of tumors (*n* = 7). **B** IHC staining of HIST3H2A. **C** IHC staining of Bax, Bcl-2,PCNA, p-RIP3 and p-MLKL in the indicated treated tumors. Scare bars, 100 μm

To further investigate the role of HIST3H2A in regulating prostate cancer progression in vivo, we conducted a xenograft tumor model in nude mice. Our results from the xenografted mice model demonstrated that the LV-HIST3H2A groups exhibited a significant decrease in tumor growth compared to the control xenografts (Fig. 5A). These findings indicate that interference with HIST3H2A inhibited the formation and growth of prostate cancer. Immunohistochemical analysis revealed that the expression of HIST3H2A and PCNA in LV-HIST3H2A tumor tissues was significantly lower than that in the control group (Fig. 5B and C). Additionally, the levels of p-RIP3 and p-MLKL were higher in the LV-HIST3H2A tumor tissue group compared to the control group, while the expression of Bax and Bcl-2 did not show significant differences (Fig. 5C). These findings suggest that HIST3H2A exerts its carcinogenic effect in vivo by inhibiting necroptosis rather than apoptosis.

## Discussion

In many parts of the world, prostate cancer is a leading cancer among men. It is the second most commonly diagnosed solid organ cancer in men, following lung cancer [42–44]. Currently, approximately 10 million men worldwide are affected by this disease, with around 700,000 having metastatic disease [45–47]. While prostate cancer is typically diagnosed at an early stage, the search for potential molecular targets for treatment is crucial due to the long history of the disease and the uncertain clinical progression of individual patients. Therefore, we conducted a screening of differentially expressed genes in prostate cancer using the GEO database to identify new molecular targets. Through this screening, the HIST3H2A gene was successfully identified, showing significantly higher expression in prostate cancer tissues and cells compared to adjacent tissues and normal prostate cells.

The nucleosome, which is the fundamental unit of chromatin, consists of approximately 146 base pairs (bp) of DNA wrapped around a single copy of each of the histone proteins H3, H4, H2A, and H2B [48–50]. Histone variations can confer different structural properties on nucleosomes by either wrapping more or less DNA or altering their stability. These histone variations perform specific functions in DNA repair, chromosome separation, transcription initiation regulation, and tissue-specific roles [51–53]. Abnormal expression of H2A family proteins has been found to cause various types of cancer in humans, such as breast, colorectal, lung, testicular, bladder, ovarian, and prostate cancers [54–56]. The HIST3H2A protein, a member of the H2A family, has been identified as a potential biomarker for pancreatic cancer, non-small cell lung cancer, glioblastoma, breast cancer, and lung cancer. However, its role in prostate cancer remains unknown. Our research findings demonstrate that HIST3H2A plays a pro-cancer role in the development and progression of prostate cancer. Overexpression of HIST3H2A can enhance cell proliferation, migration, invasion, and promote the epithelial-mesenchymal transition (EMT) process in tumors. Conversely, interfering with HIST3H2A expression leads to the opposite effect.

necroptosis is a regulated form of cell death that differs from apoptosis as it does not depend on caspase [57, 58]. necroptosis requires RIPK3, which activates RIPK3 and leads to an increase in phosphorylated MLKL [59]. This phosphorylated MLKL then oligomerizes to form activated ‘bad dead’ complexes and translocates to the plasma membrane [60, 61]. This process ultimately results in cell death, characterized by plasma membrane penetration, cell swelling, and loss of cell and organelle integrity [62–64]. The significance of necroptosis in cancer is increasingly recognized, and a better understanding of this process may contribute to the development of new cancer control strategies. To further investigate the role of HIST3H2A in regulating the progression of prostate cancer, we conducted correlation analysis using the KEGG database. The results revealed that HIST3H2A is involved in regulating the necroptosis signaling pathway. Additionally, western blot and JC-1 tests demonstrated that HIST3H2A regulates the biology and function of prostate cancer cells through necroptosis rather than apoptosis.

To further confirm the role of HIST3H2A in vivo, we conducted xenograft tumor experiments in nude mice. The findings revealed that interfering with HIST3H2A significantly inhibited tumor growth in these mice. Additionally, IHC tests demonstrated that HIST3H2A inhibits the proliferation of prostate cancer cells in vivo by promoting necroptosis instead of apoptosis. In conclusion, our results suggest that HIST3H2A promotes the progression of prostate cancer by inhibiting cell necroptosis rather than apoptosis. Our study provides new potential molecular targets for the treatment of prostate cancer.

### Electronic supplementary material

Below is the link to the electronic supplementary material.

Supplementary Material 1

Supplementary Material 2

Supplementary Material 3

## Data Availability

The datasets used and/or analysed during the current study available from the corresponding author on reasonable request.
