# Supplementary material for: HIST3H2A promotes the progression of prostate cancer through inhibiting cell necroptosis
Source: BMC Cancer. 2024 Apr 29;24:544. doi: 10.1186/s12885-024-12308-4 (PMC11059659; doi:10.1186/s12885-024-12308-4)

Fig. 1 F (HIST3H2A)

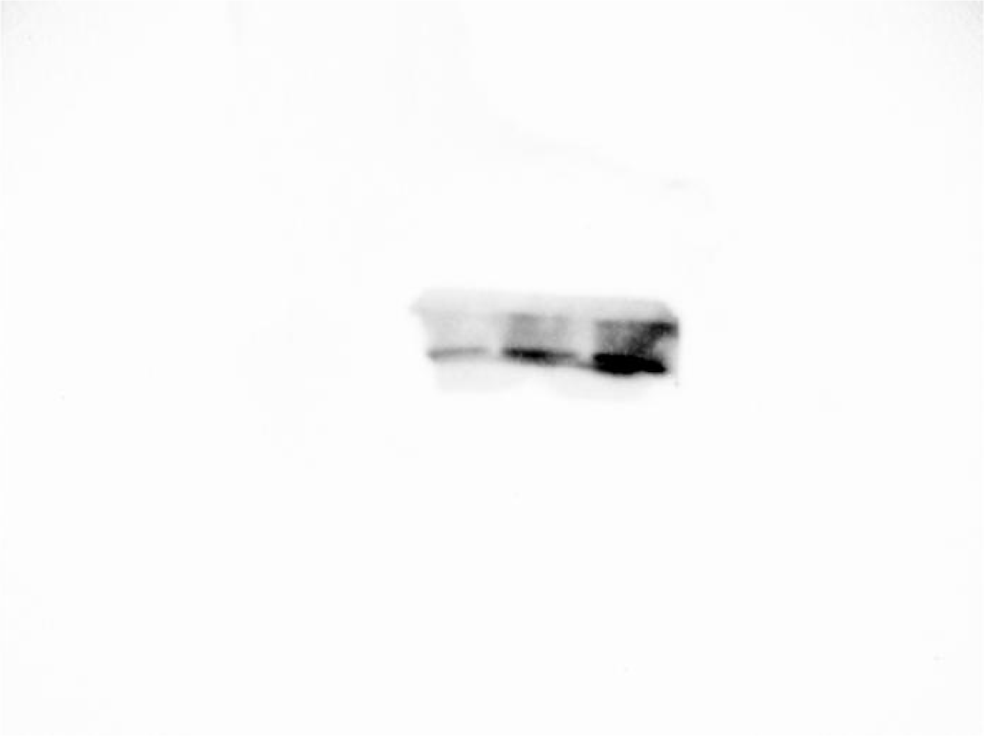

Fig. 1 F (GAPDH)

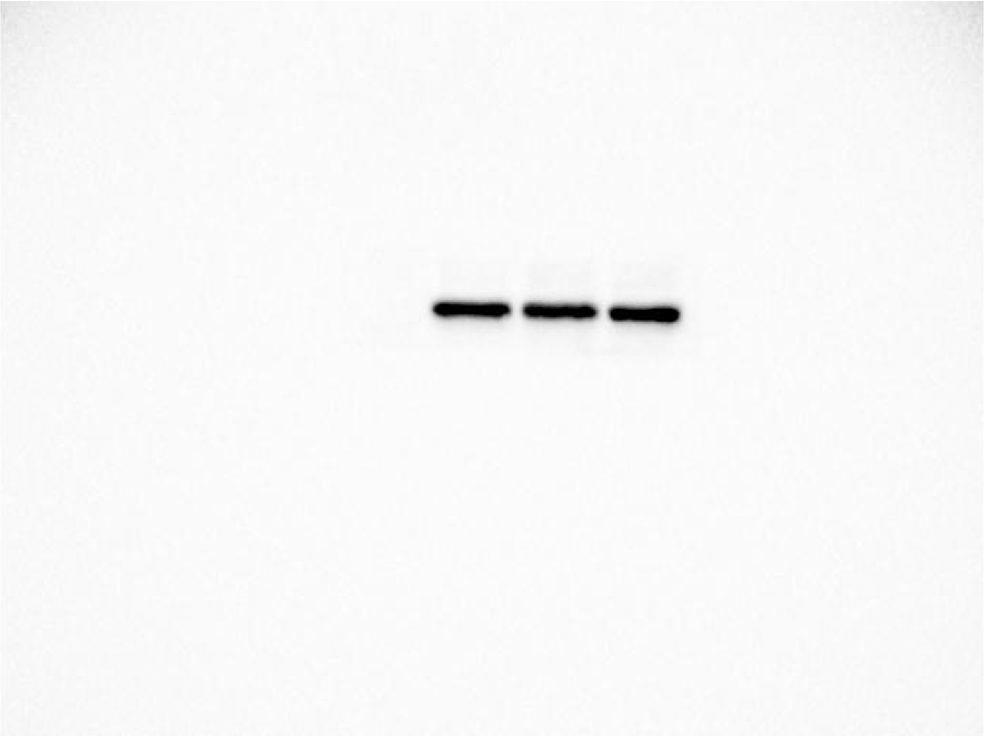

Fig. 2 A (HIST3H2A)

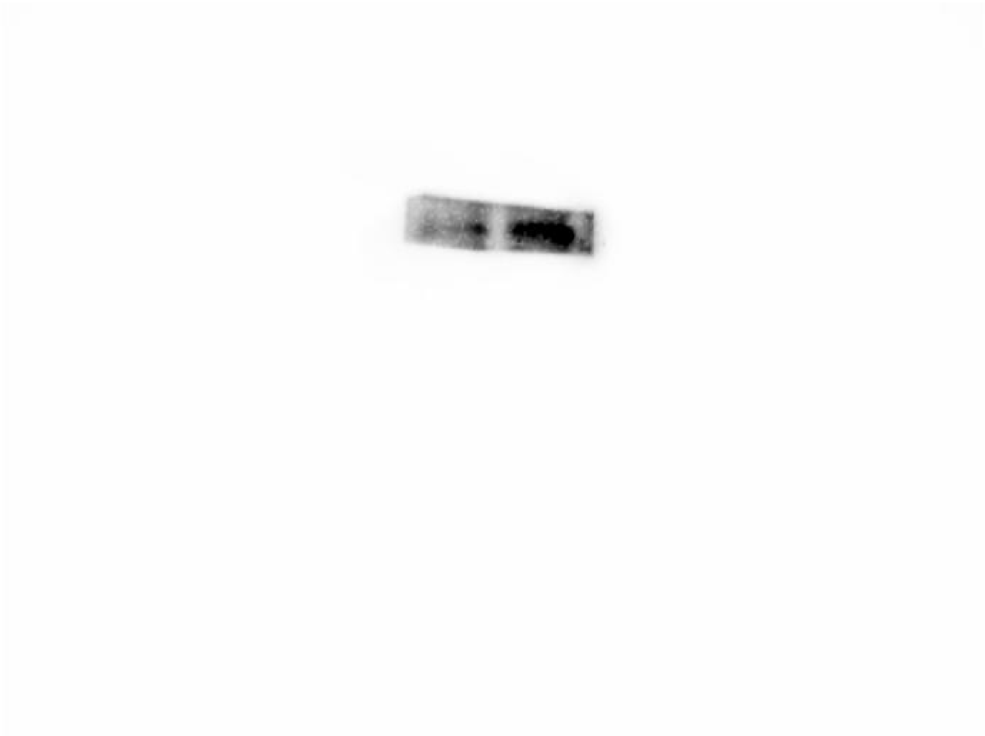

Fig. 2 A (GAPDH)

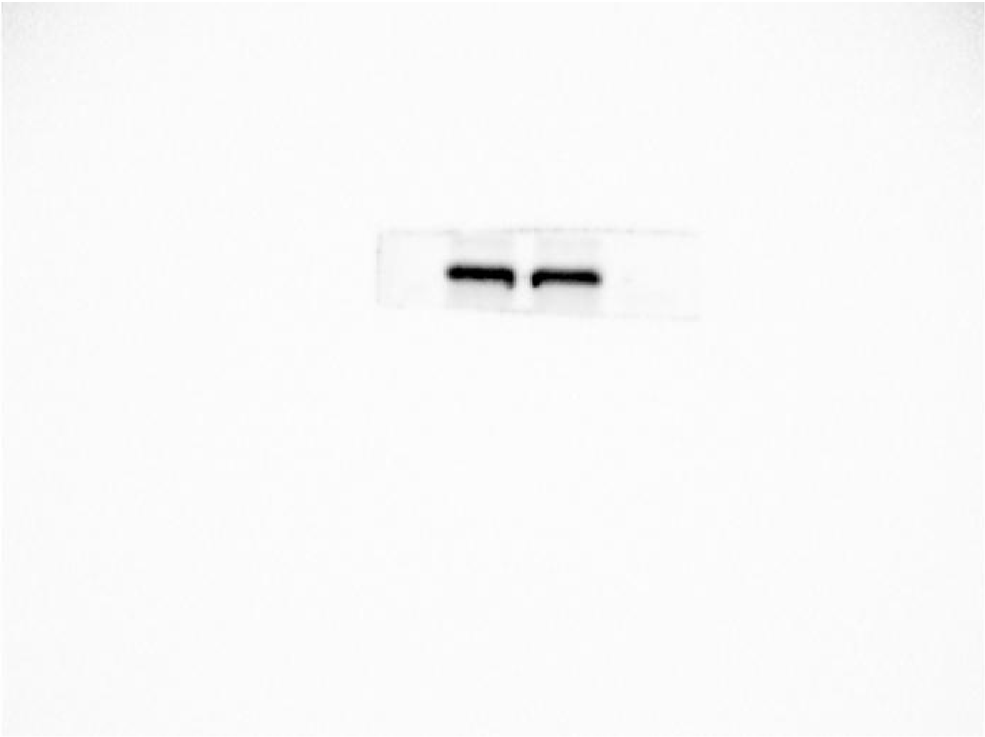

Fig. 2 B (HIST3H2A)

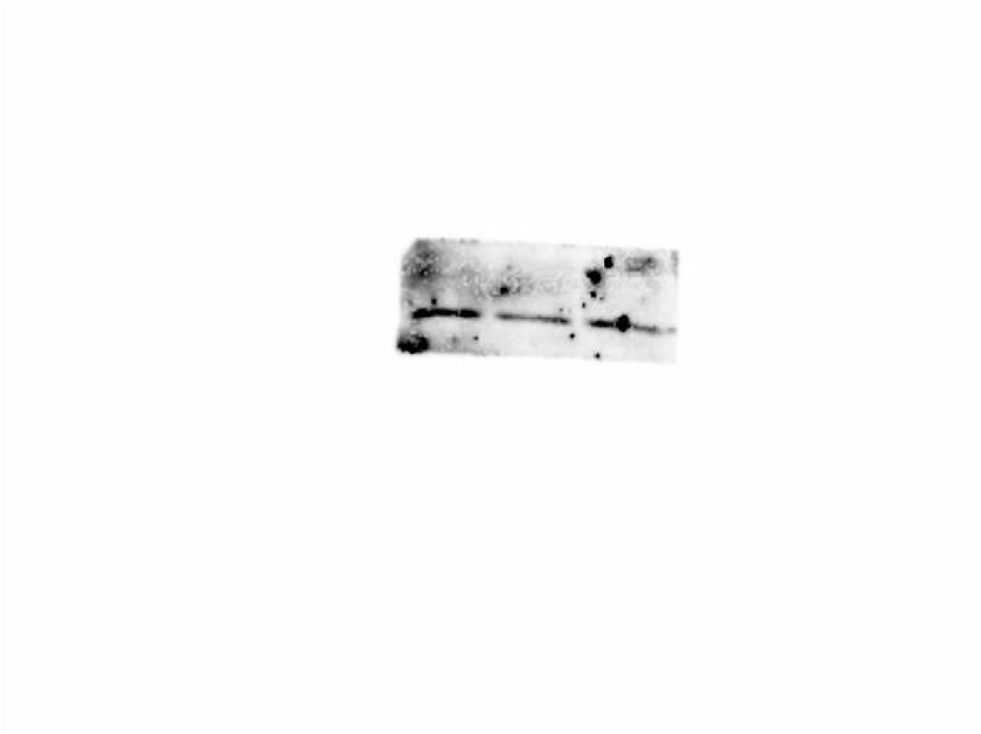

Fig. 2 B (GAPDH)

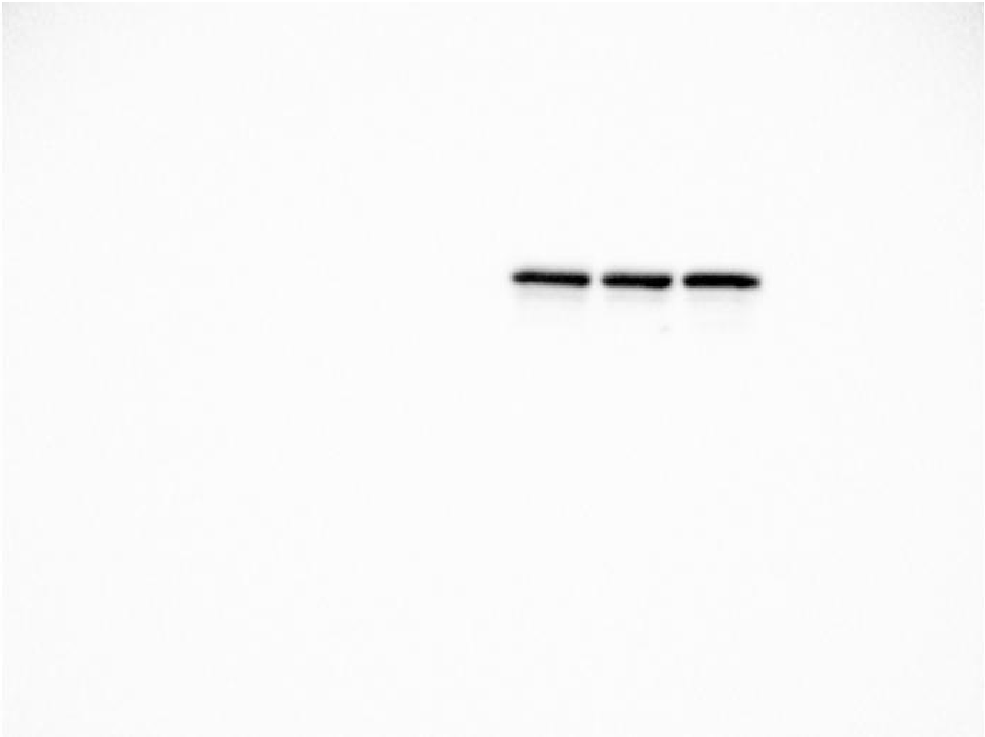

Fig. 2 C (PCNA)

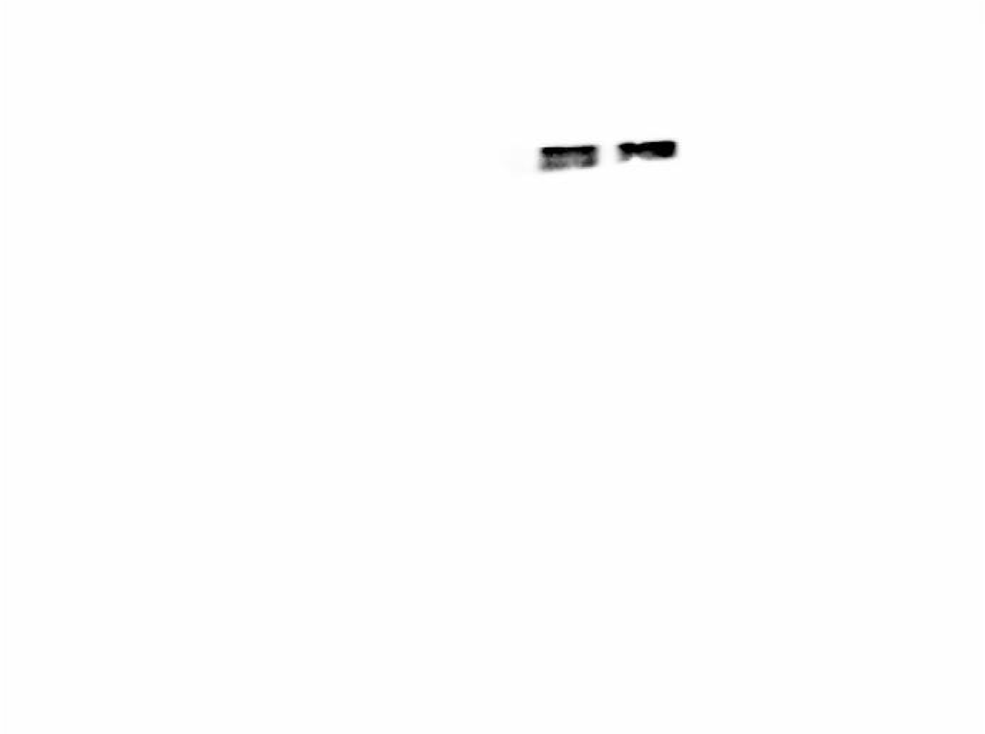

Fig. 2 C (GAPDH)

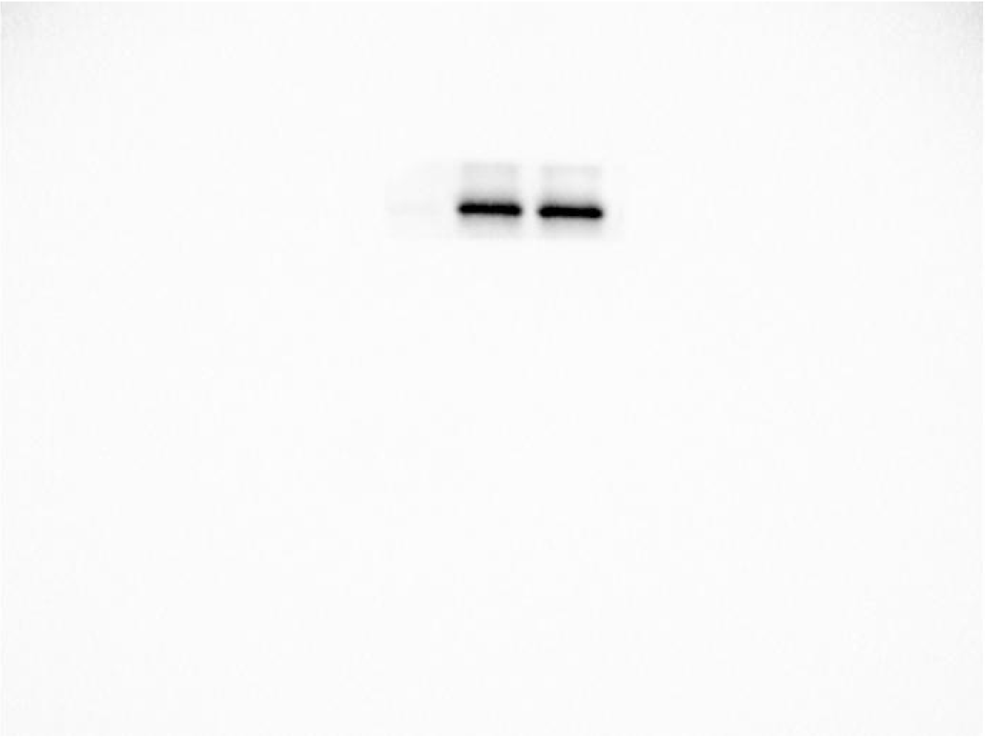

Fig. 2 D (PCNA)

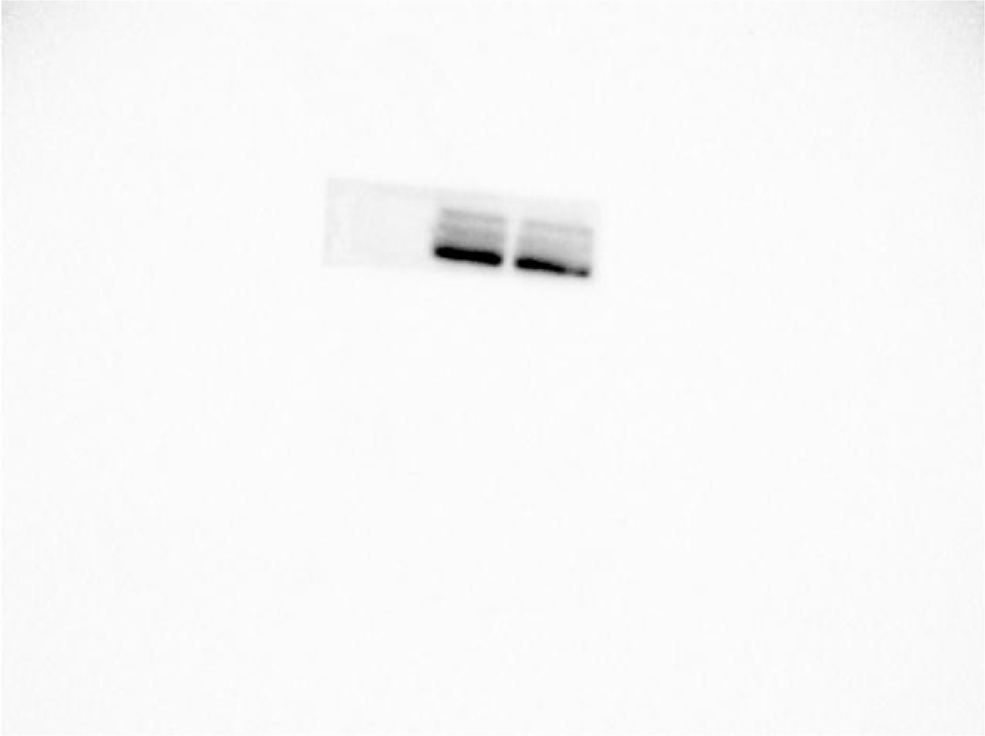

Fig. 2 D (GAPDH)

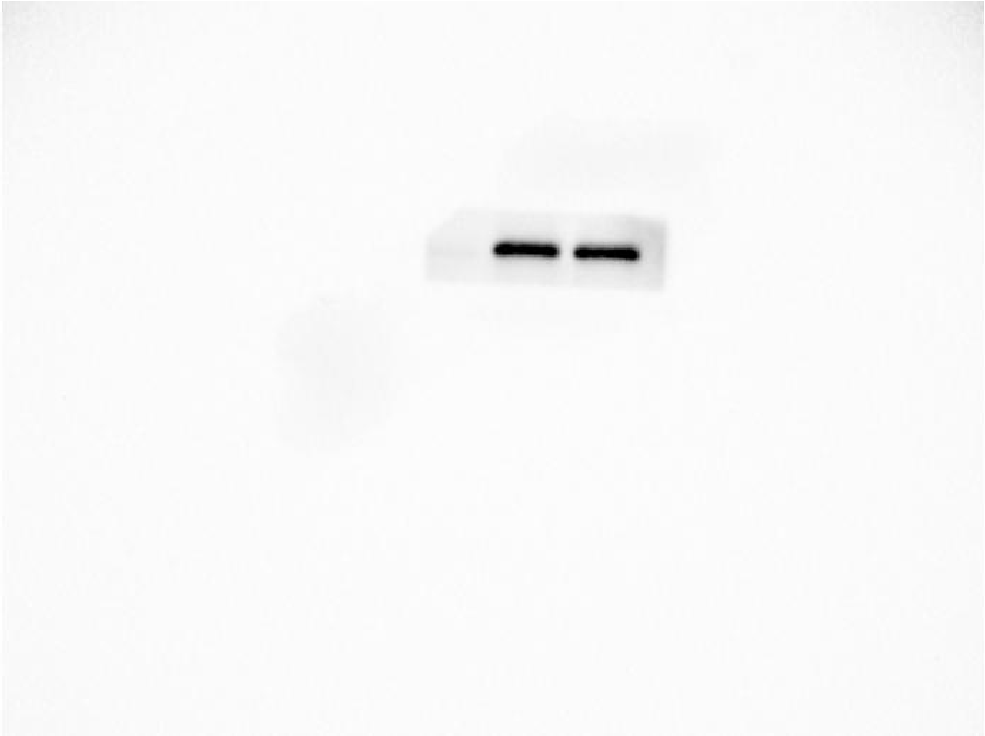

Fig. 2 E (CDK2)

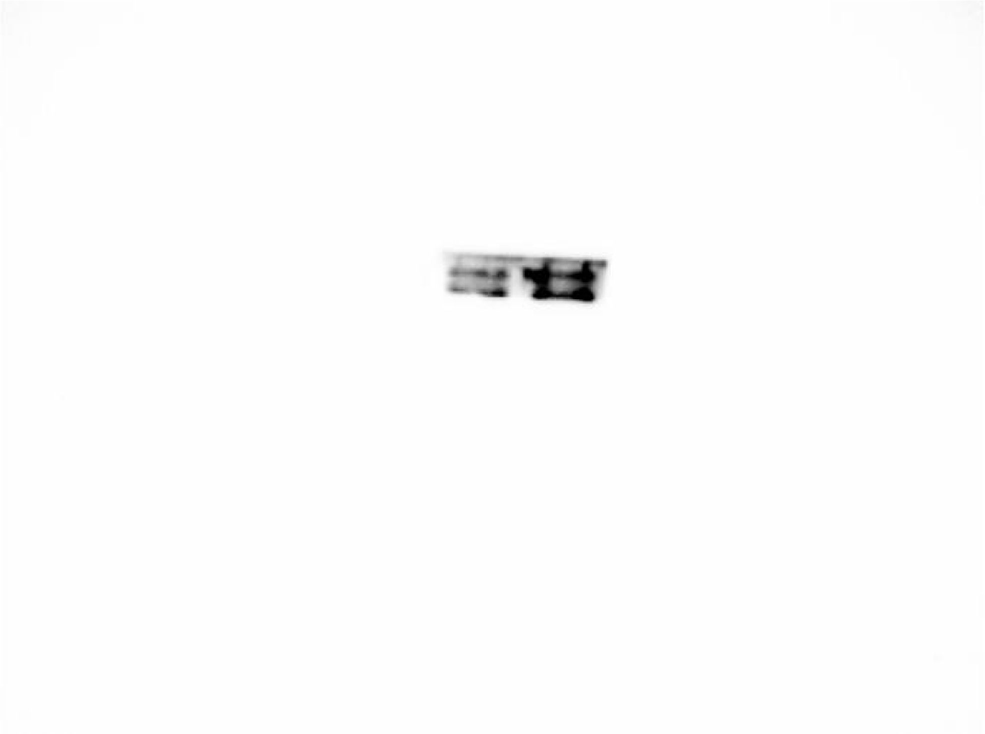

Fig. 2 E (CDK6)

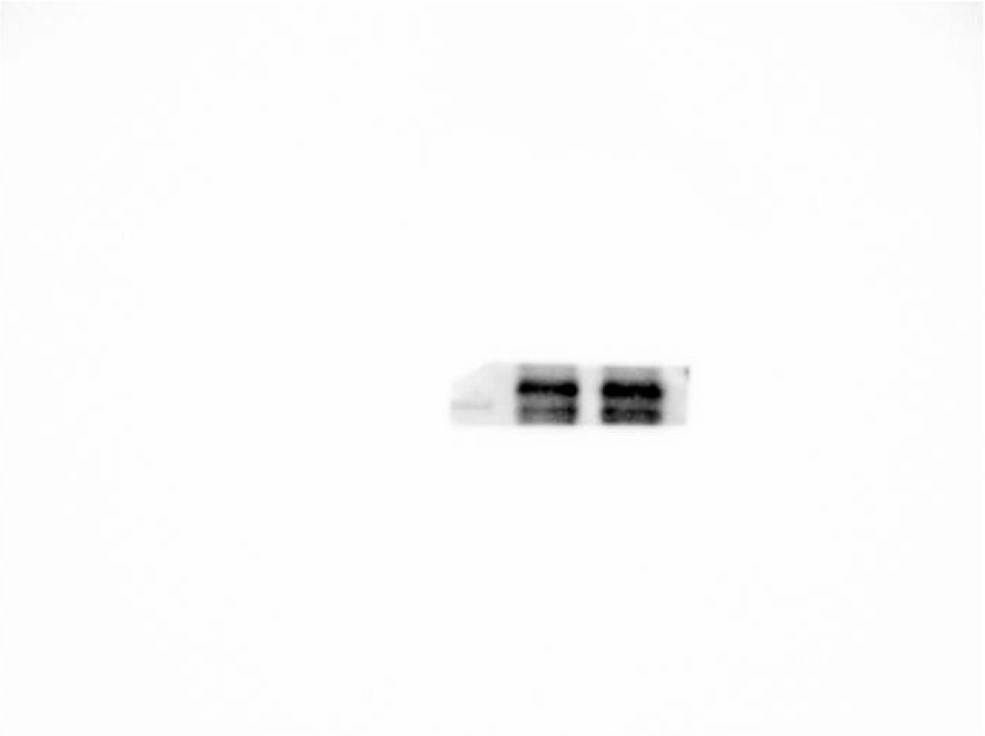

Fig. 2 E (CyclinD1)

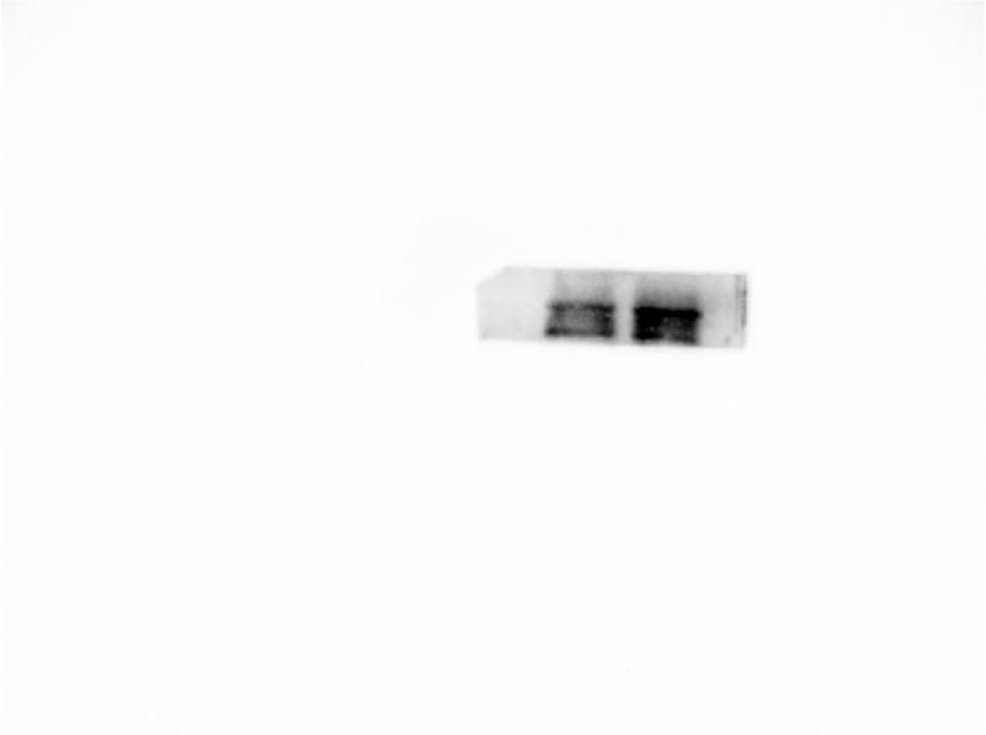

Fig. 2 E (CyclinE1)

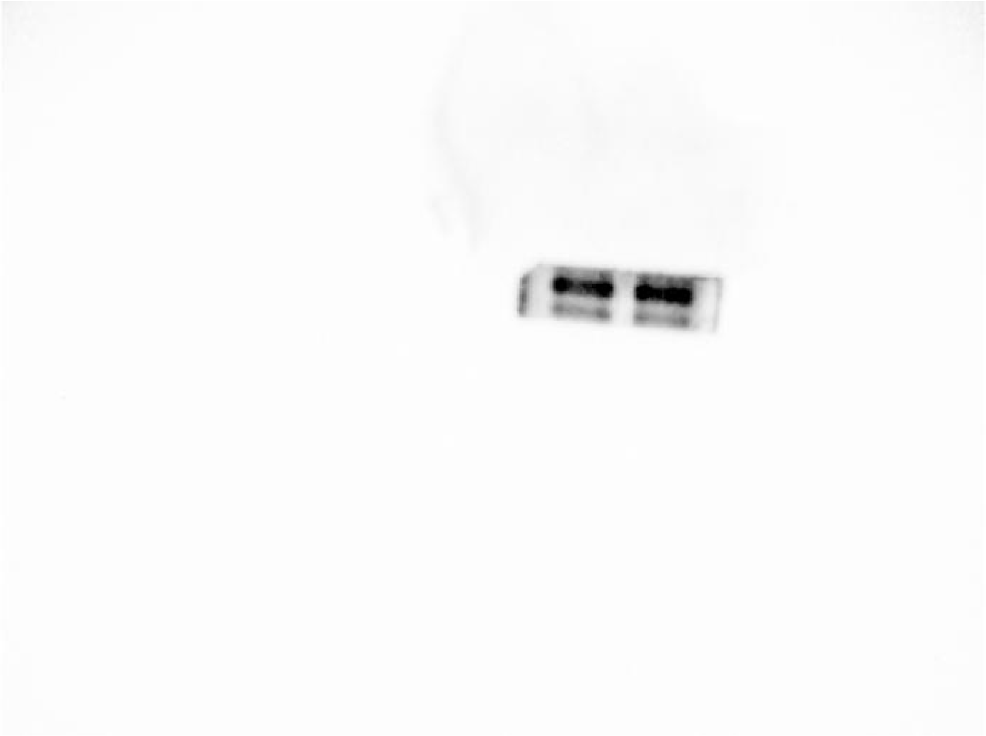

Fig. 2 E (P21)

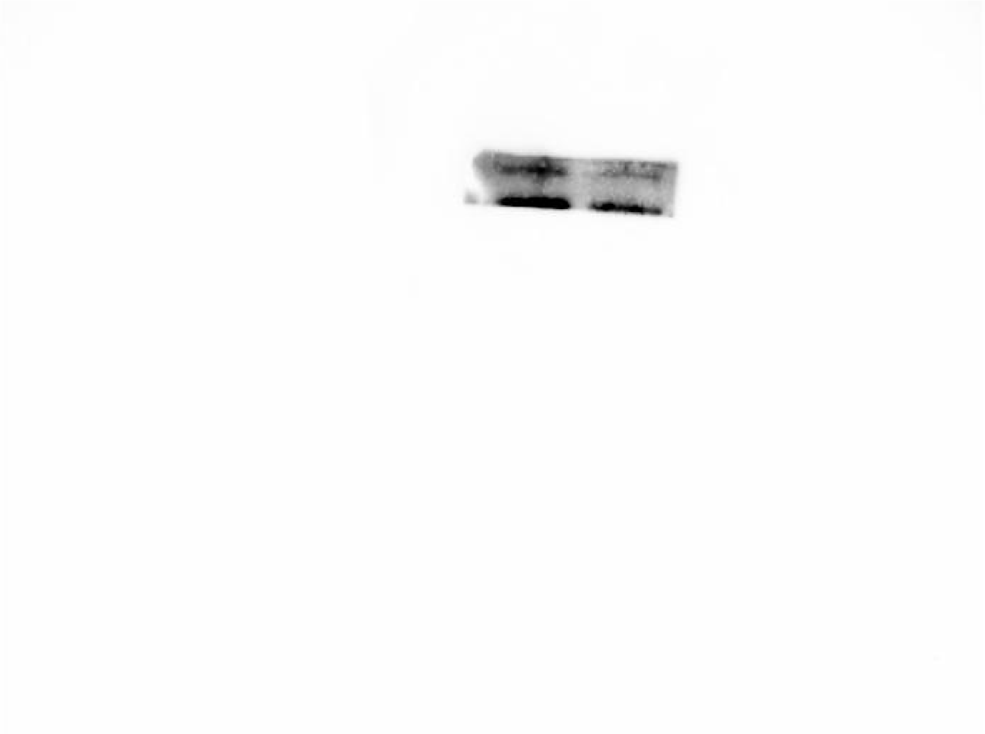

Fig. 2 E (GAPDH)

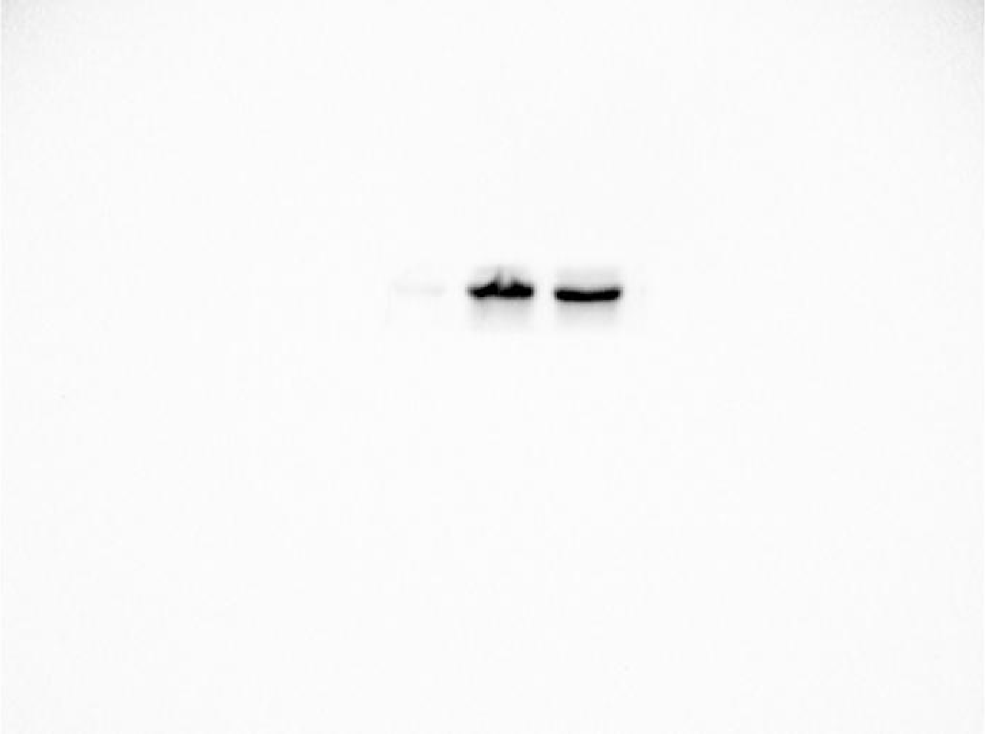

Fig. 2 F (CDK2)

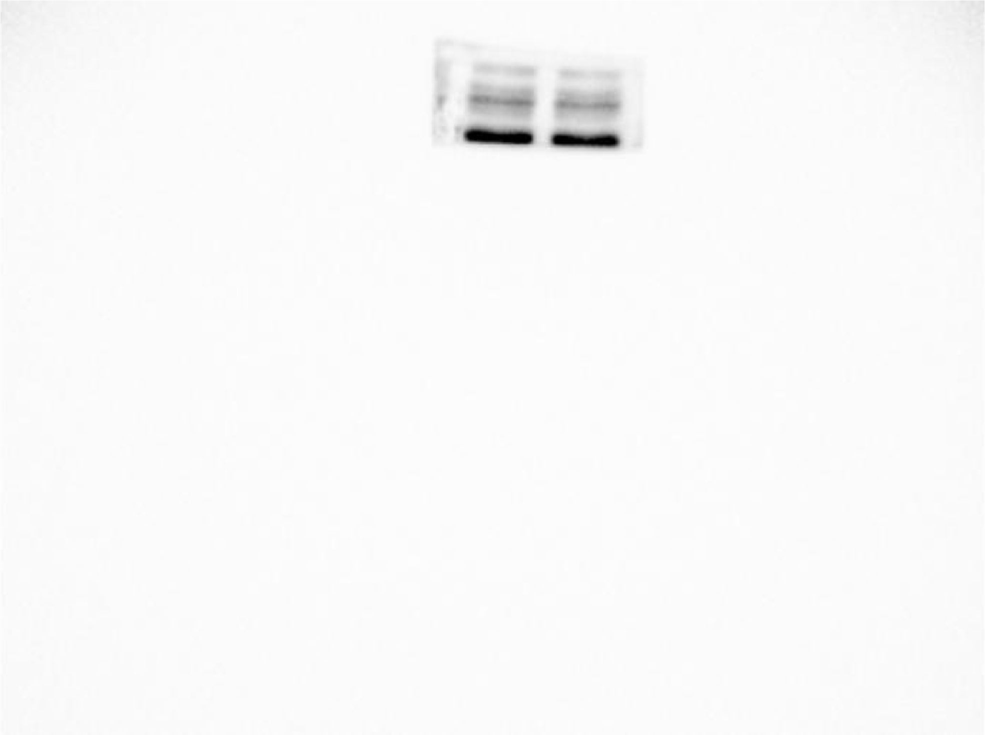

Fig. 2 F (CDK6)

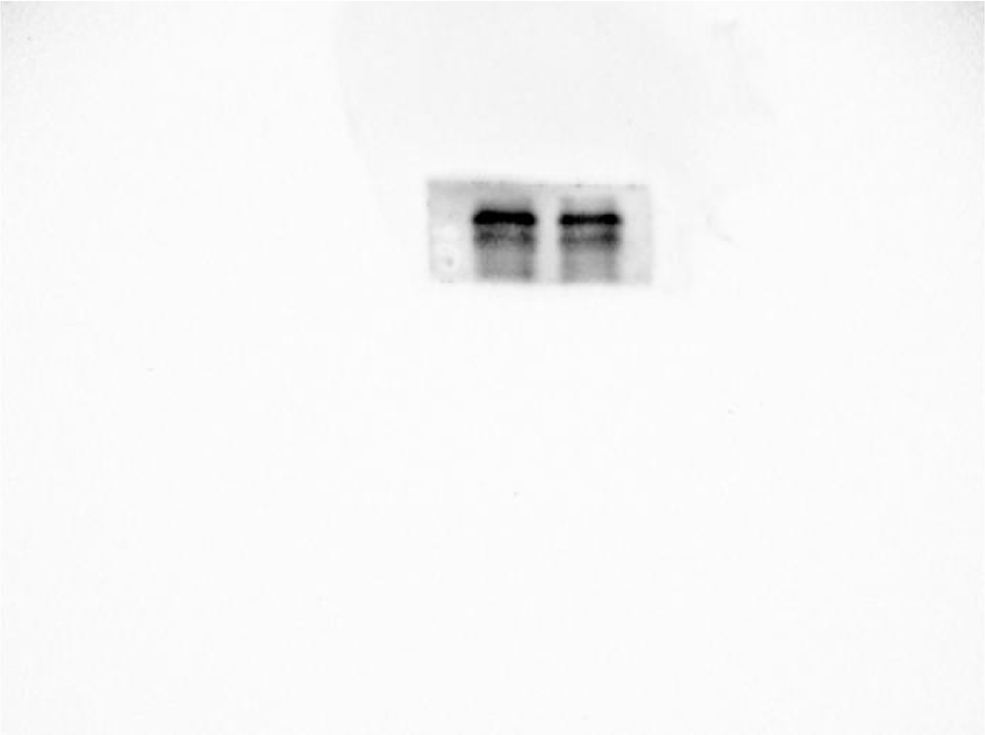

Fig. 2 F (CyclinD1)

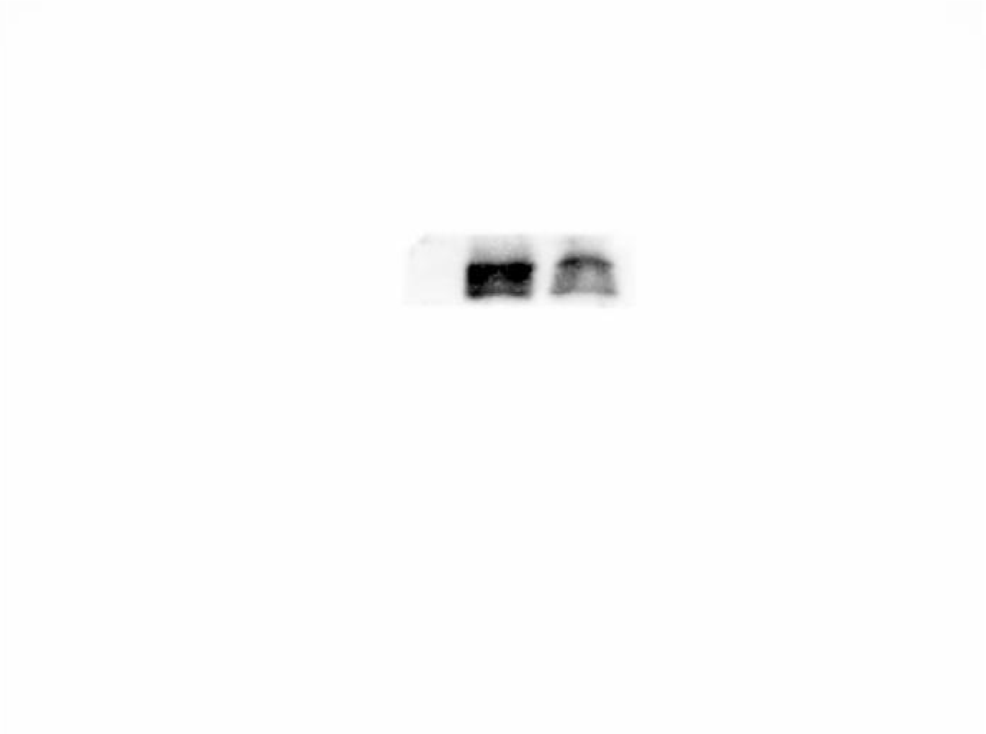

Fig. 2 F (CyclinE1)

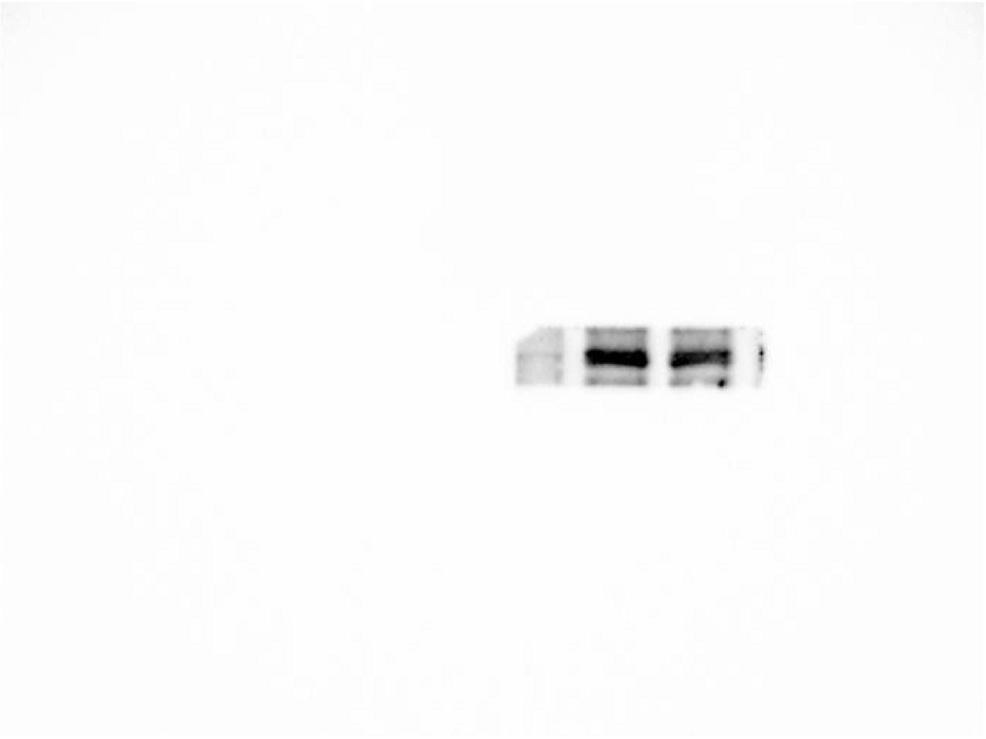

Fig. 2 F (P21)

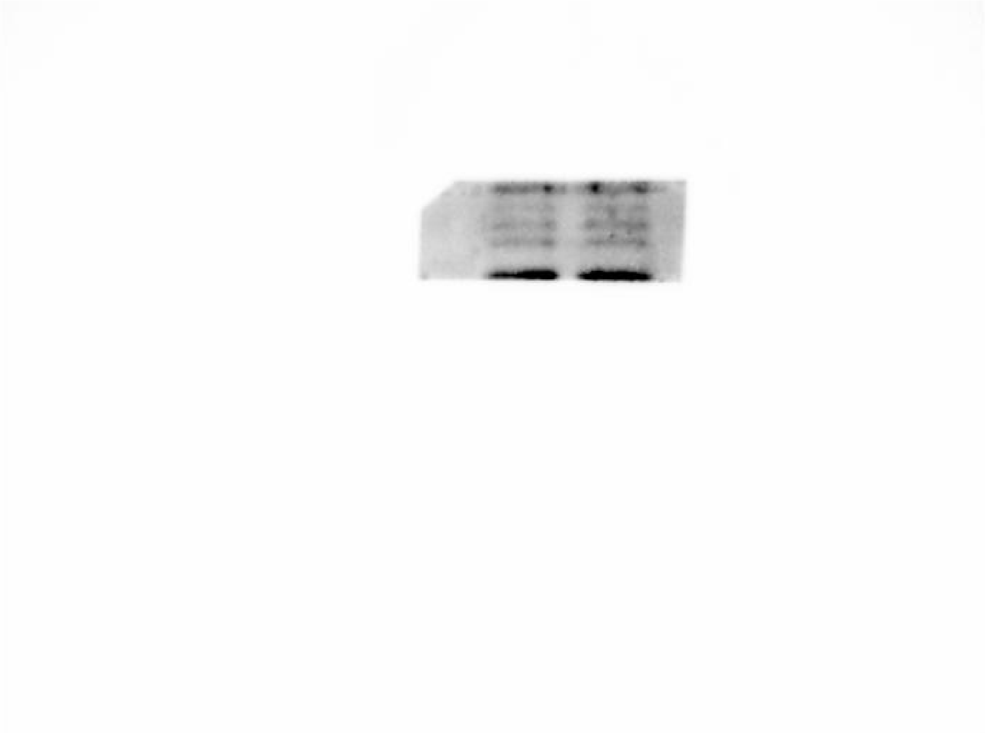

Fig. 2 F (GAPDH)

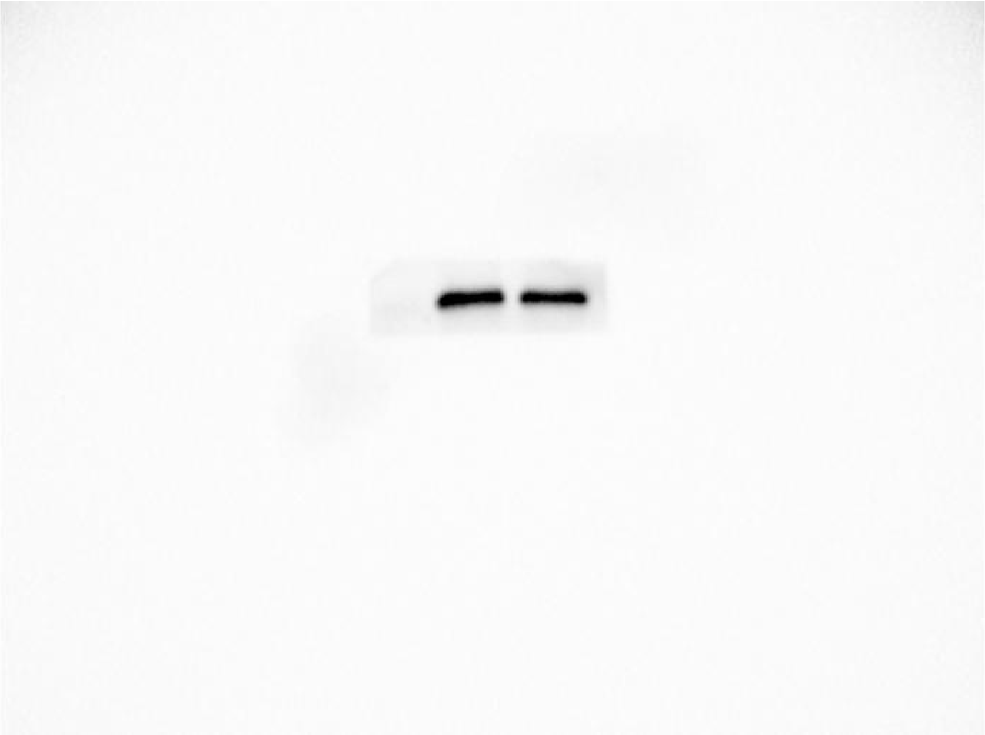

Fig. 3 A (MMP-2)

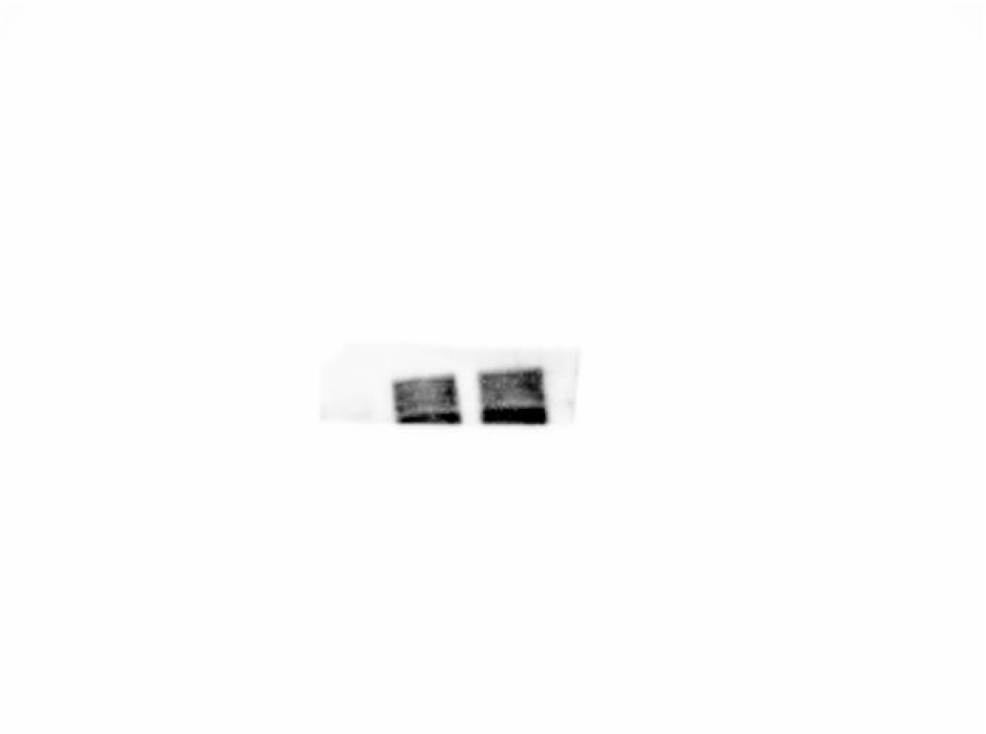

Fig. 3 A (MMP-9)

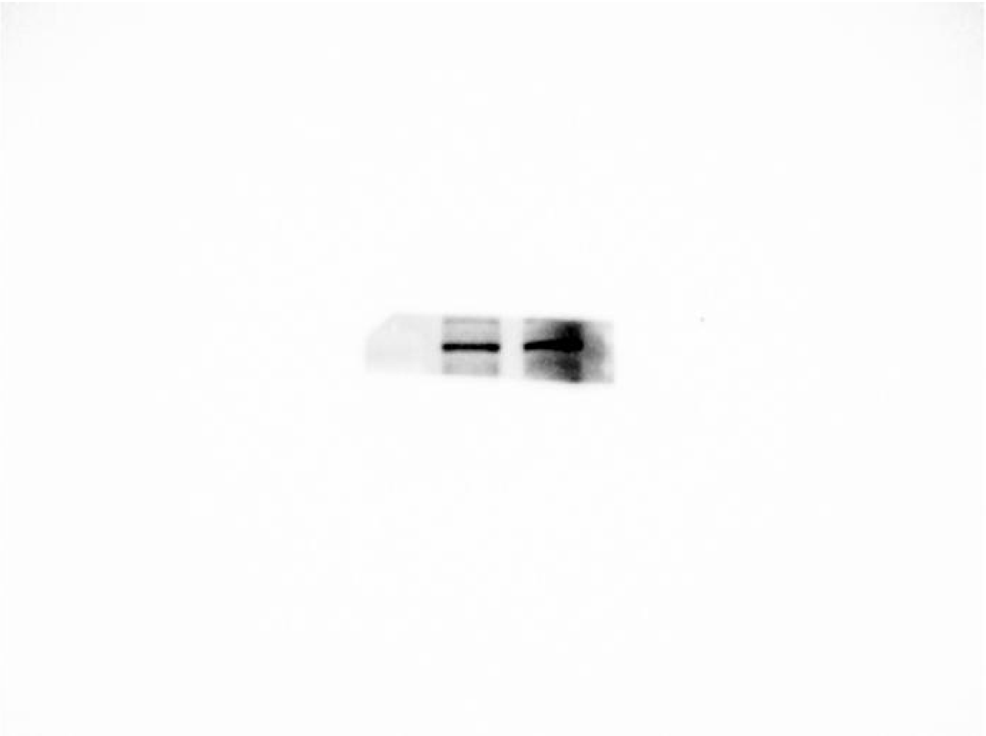

Fig. 3 A (GAPDH)

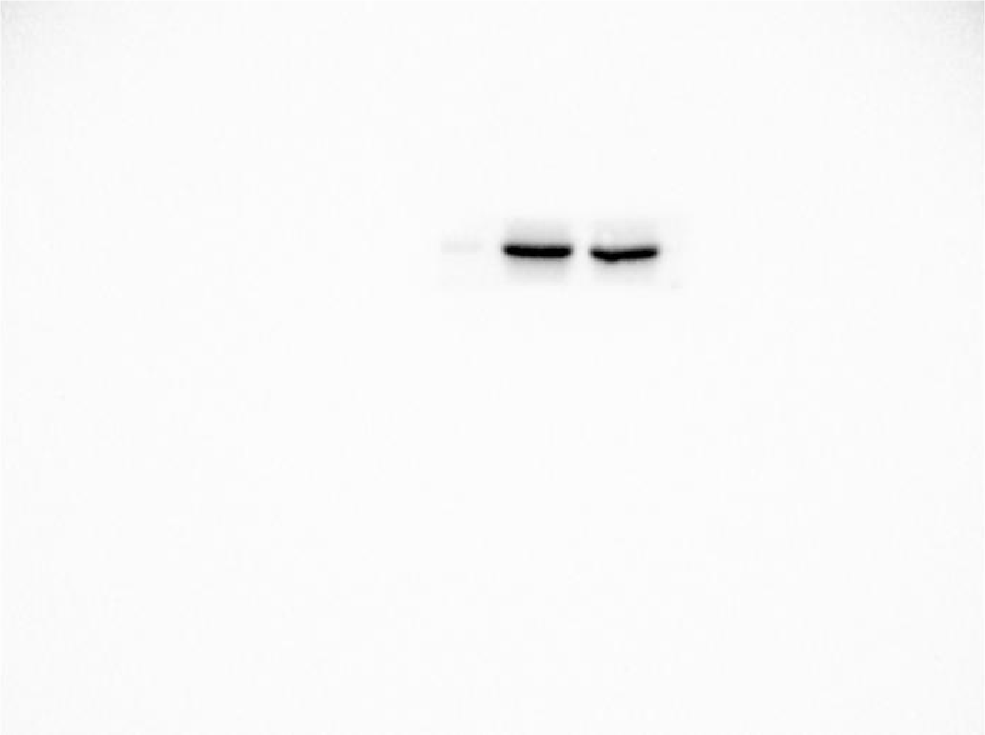

Fig. 3 B (MMP-2)

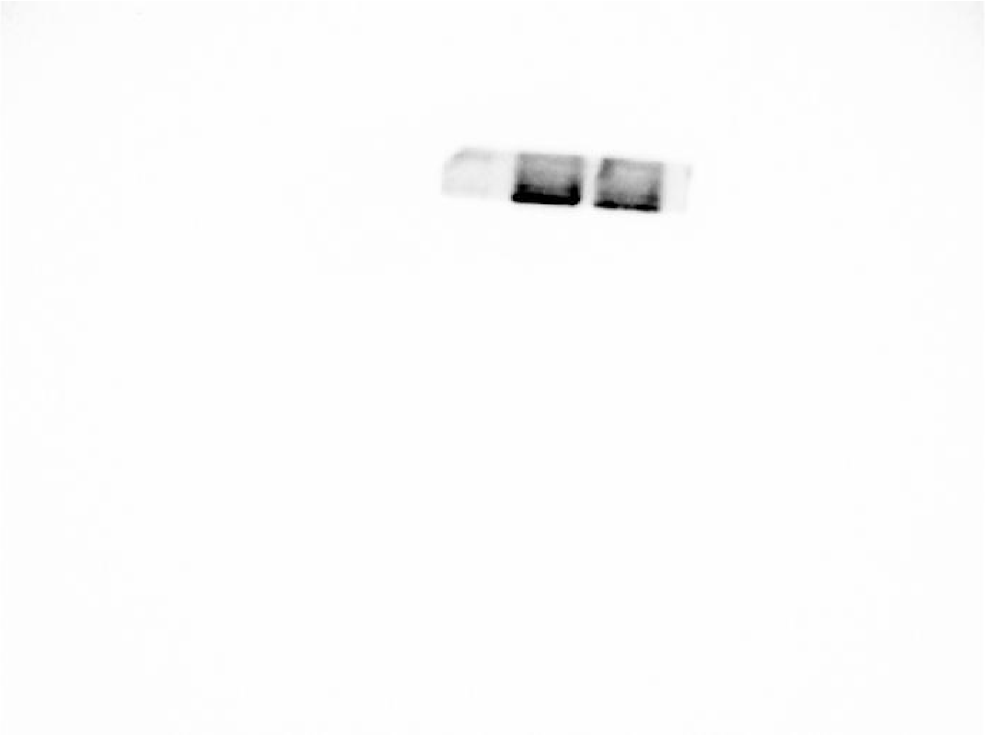

Fig. 3 B (MMP-9)

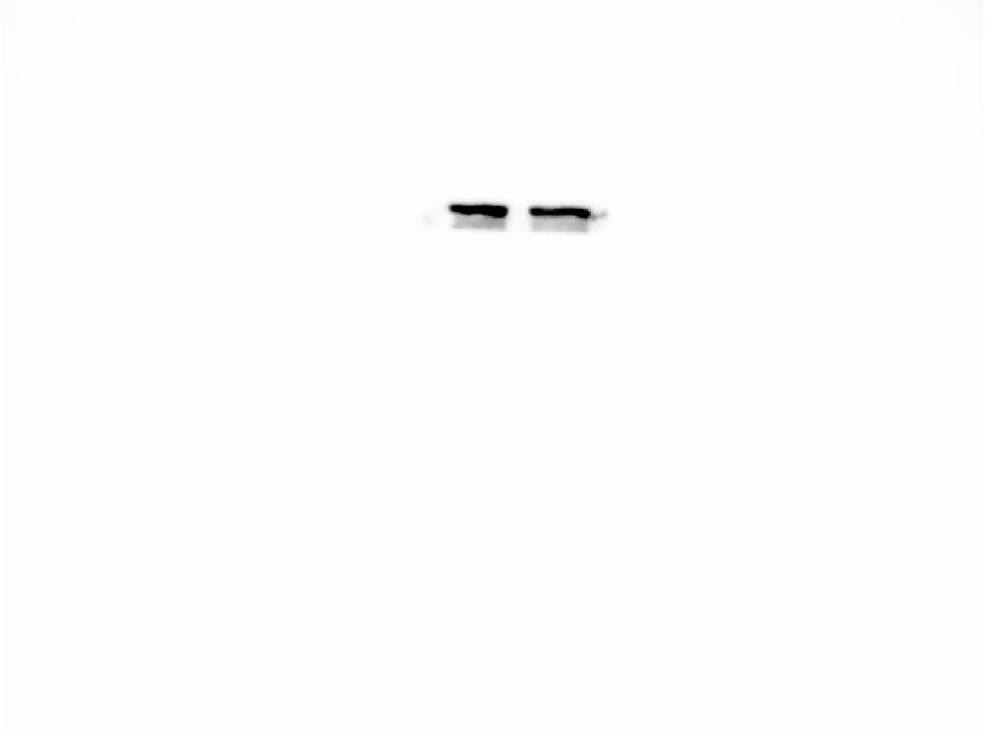

Fig. 3 B (GAPDH)

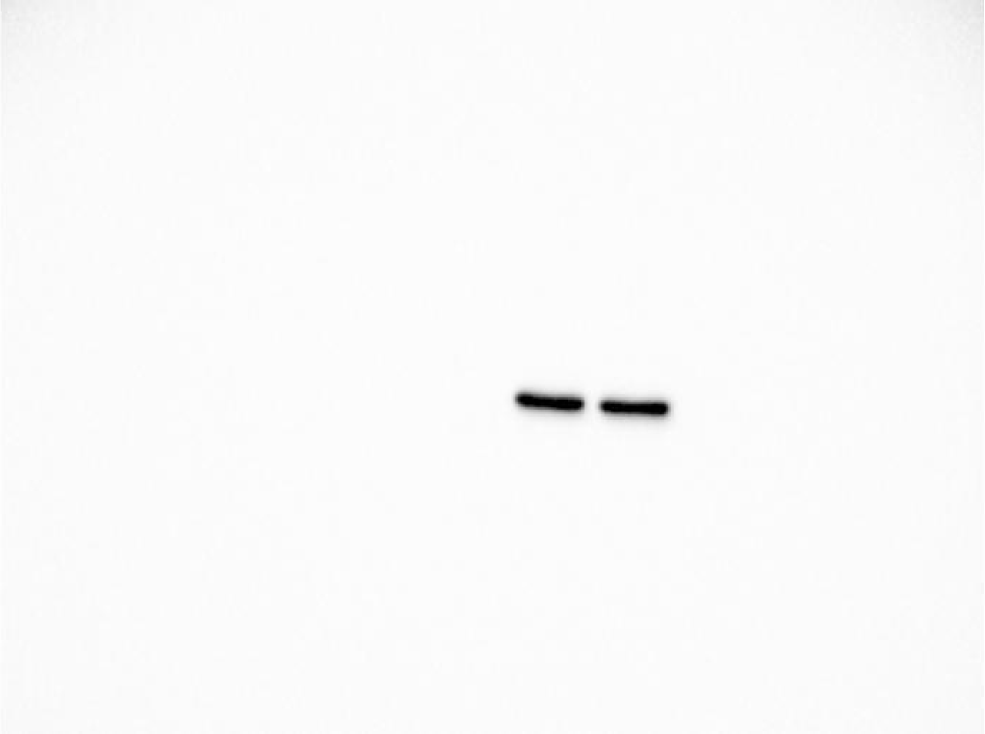

Fig. 3 E (E-cadherin)

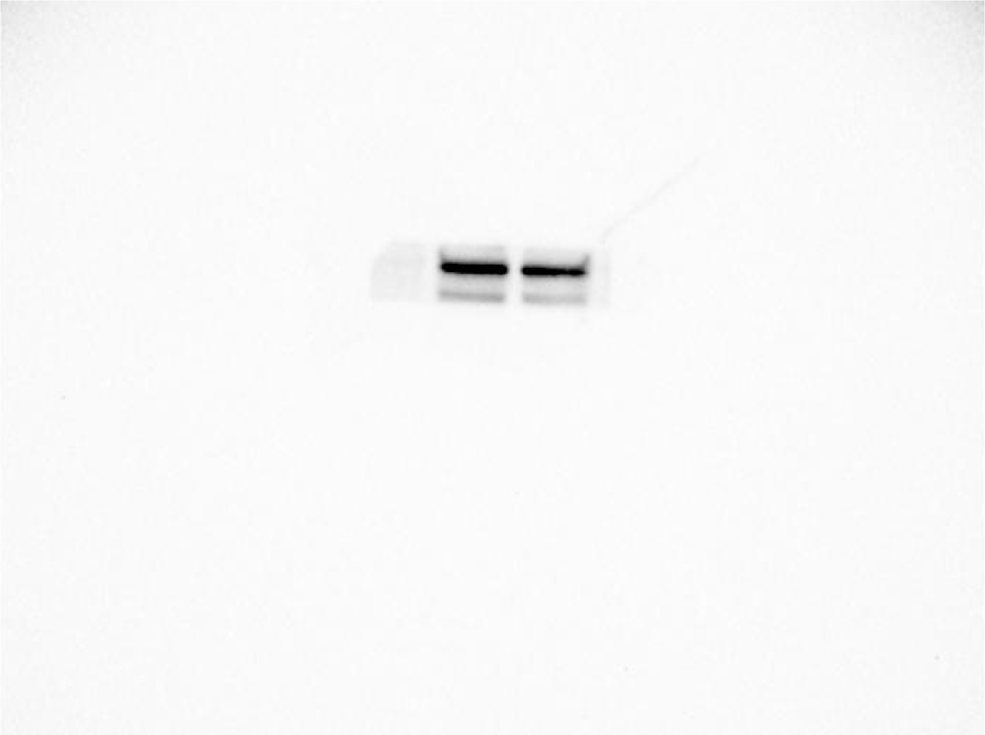

Fig. 3 E (N-cadherin)

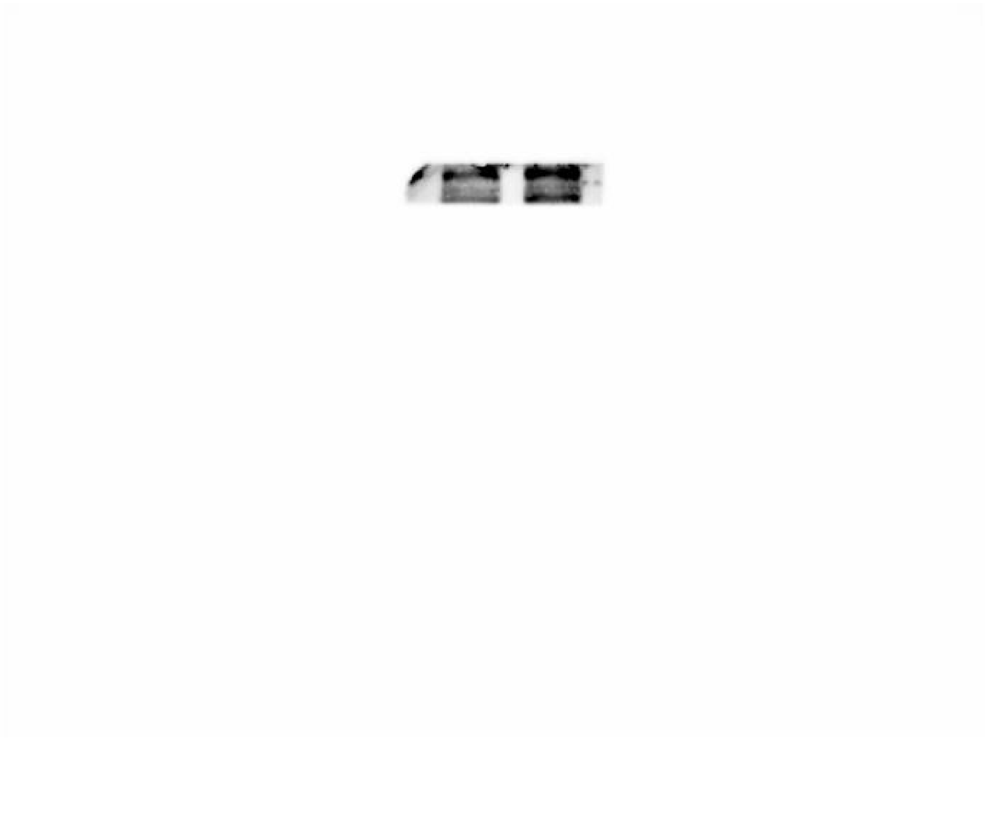

Fig. 3 E (Vimentin)

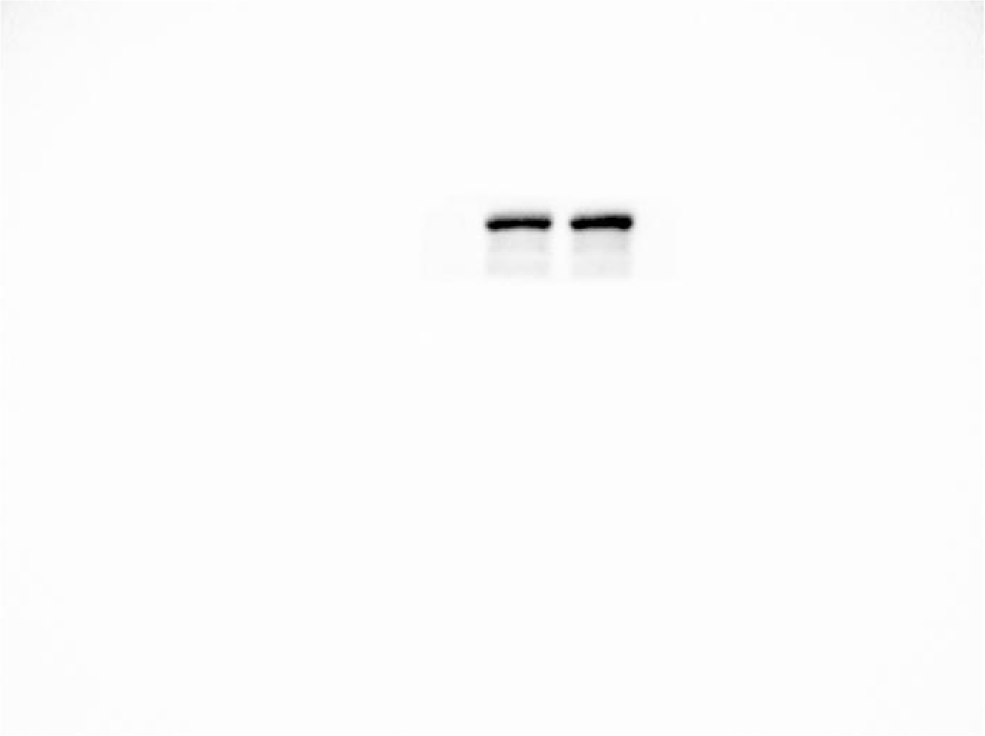

Fig. 3 E (GAPDH)

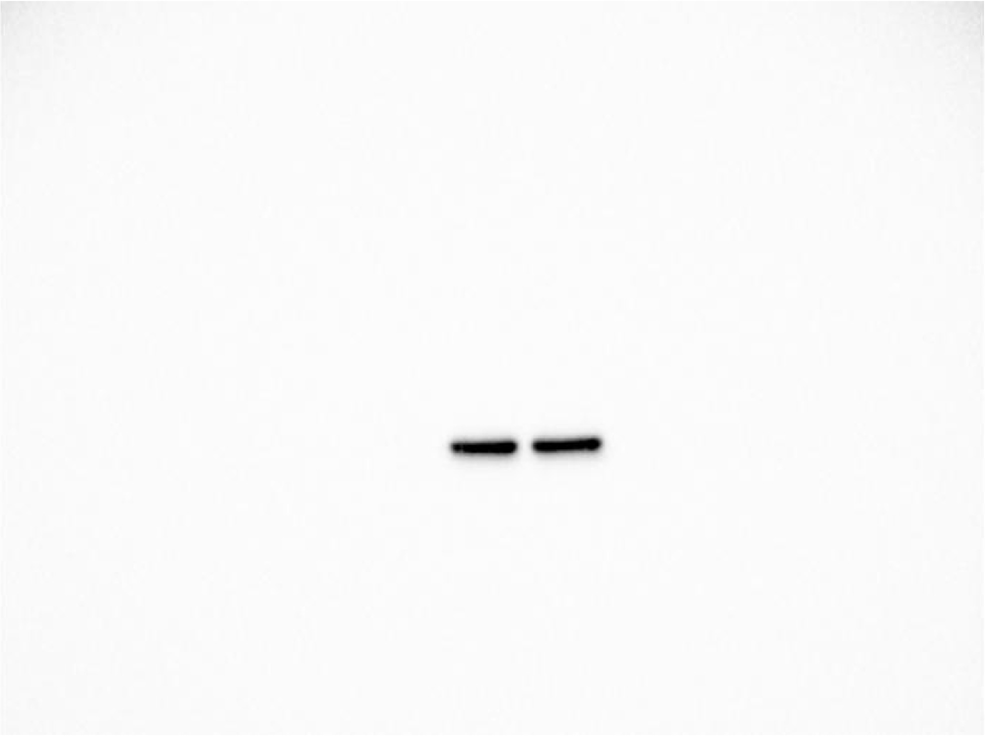

Fig. 3 F (E-cadherin)

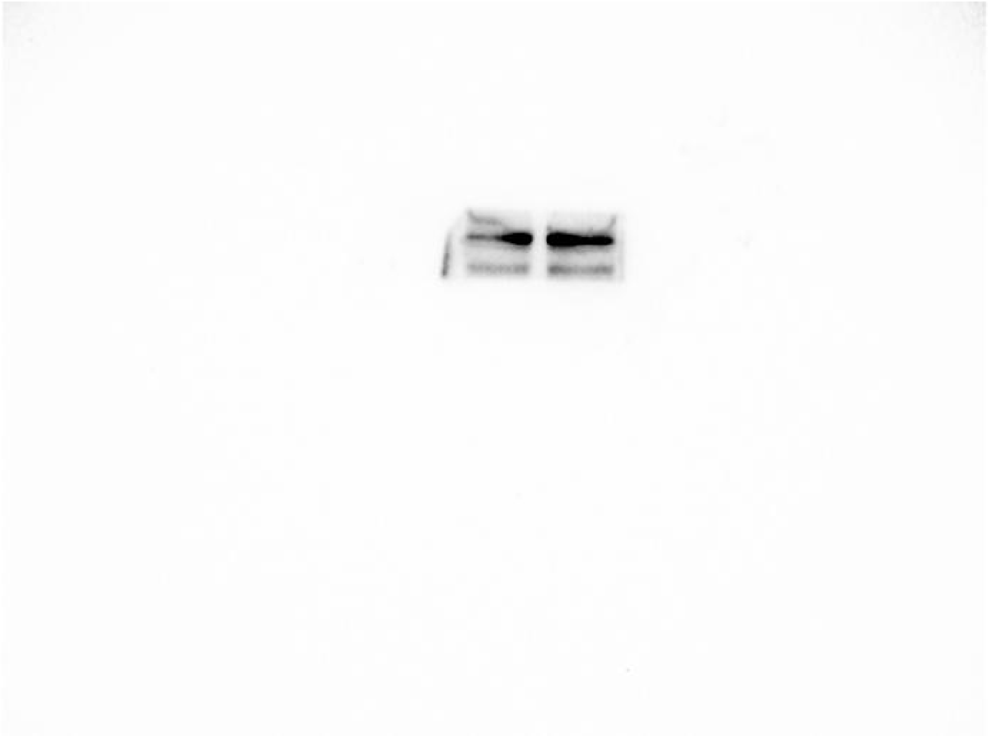

Fig. 3 F (N-cadherin)

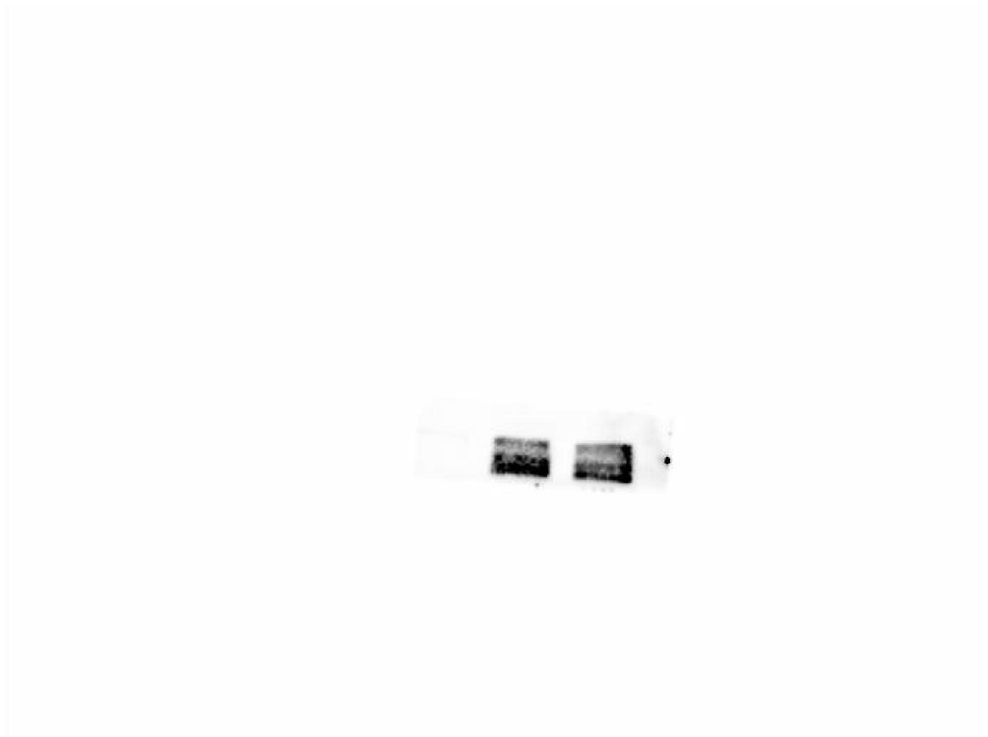

Fig. 3 F (Vimentin)

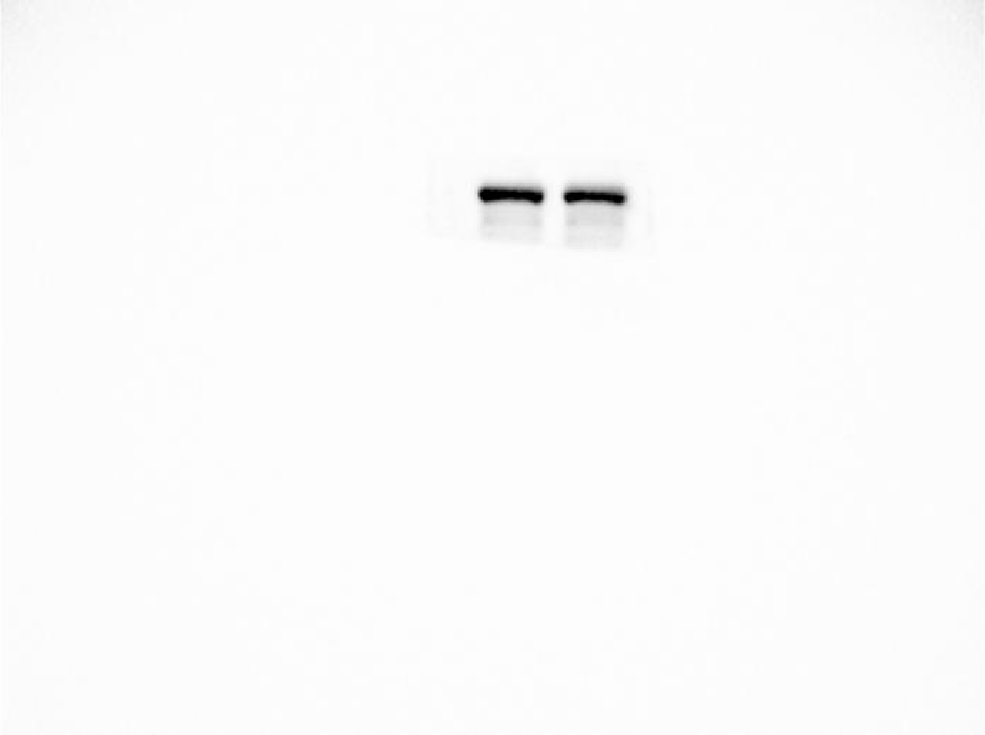

Fig. 3 F (GAPDH)

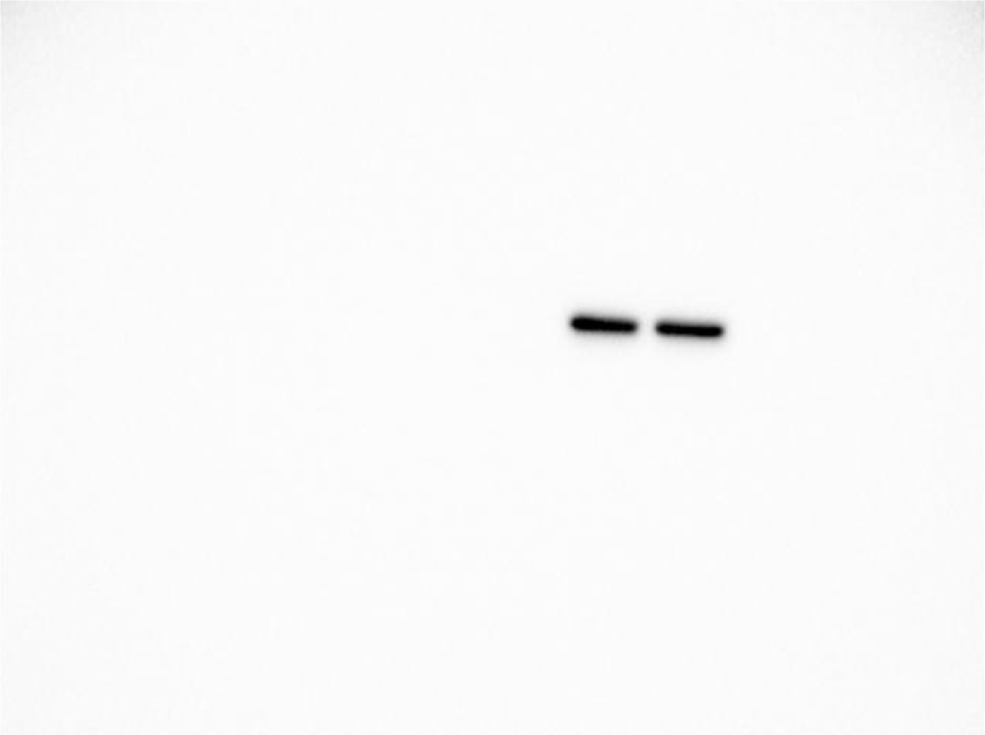

Fig. 4 A (Bcl-2)

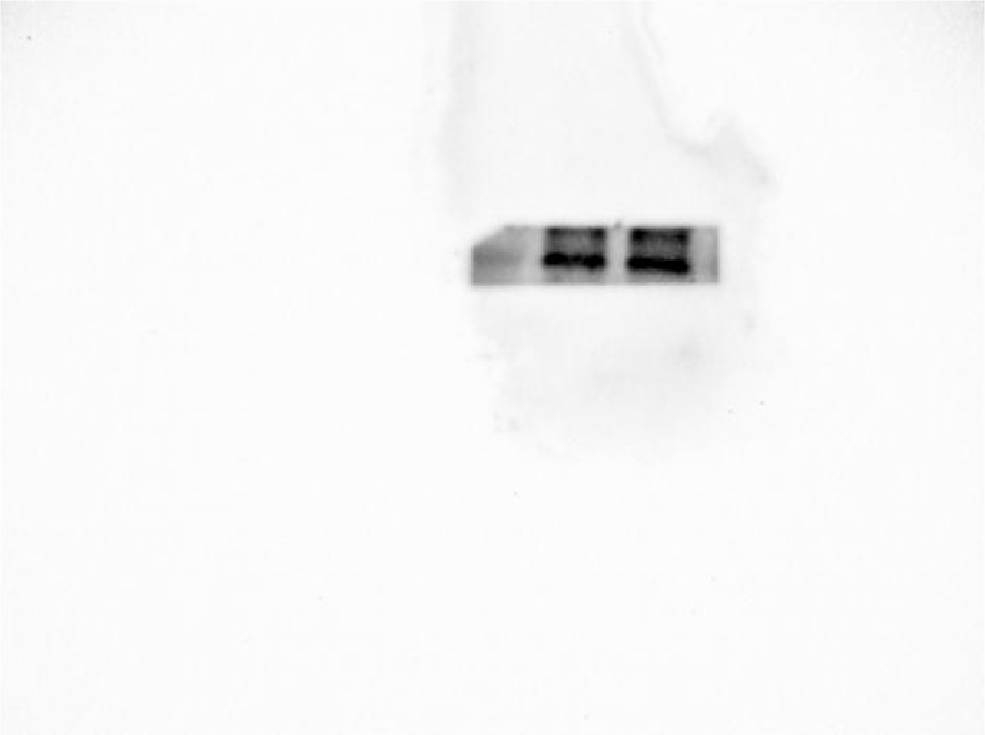

Fig. 4 A (BAX)

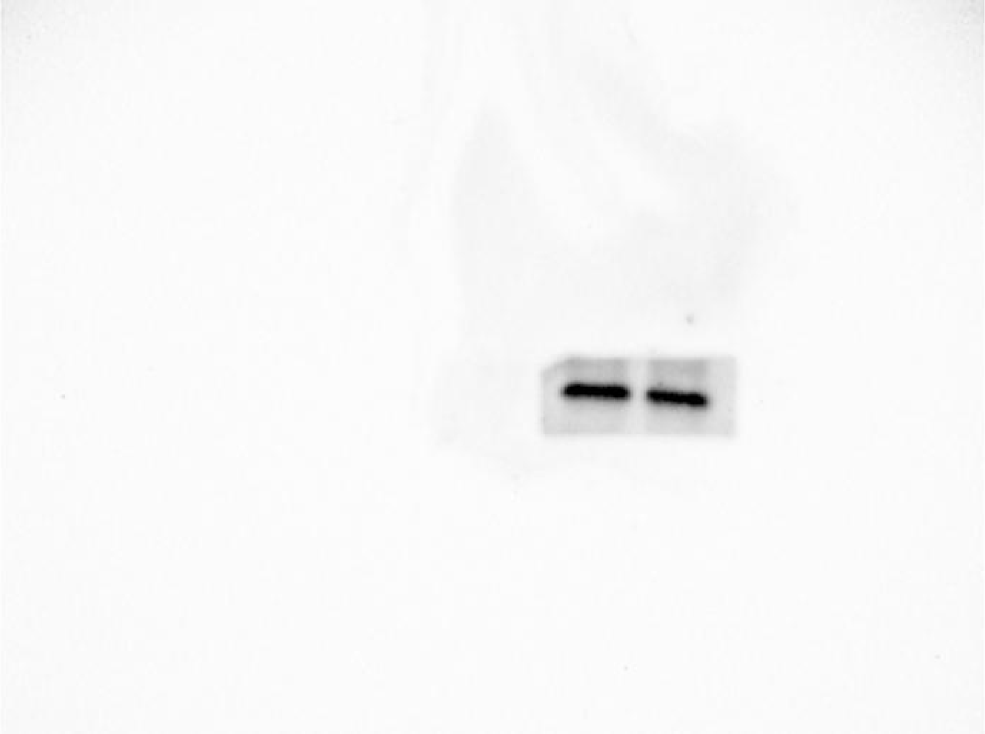

Fig. 4 A (Caspase-9)

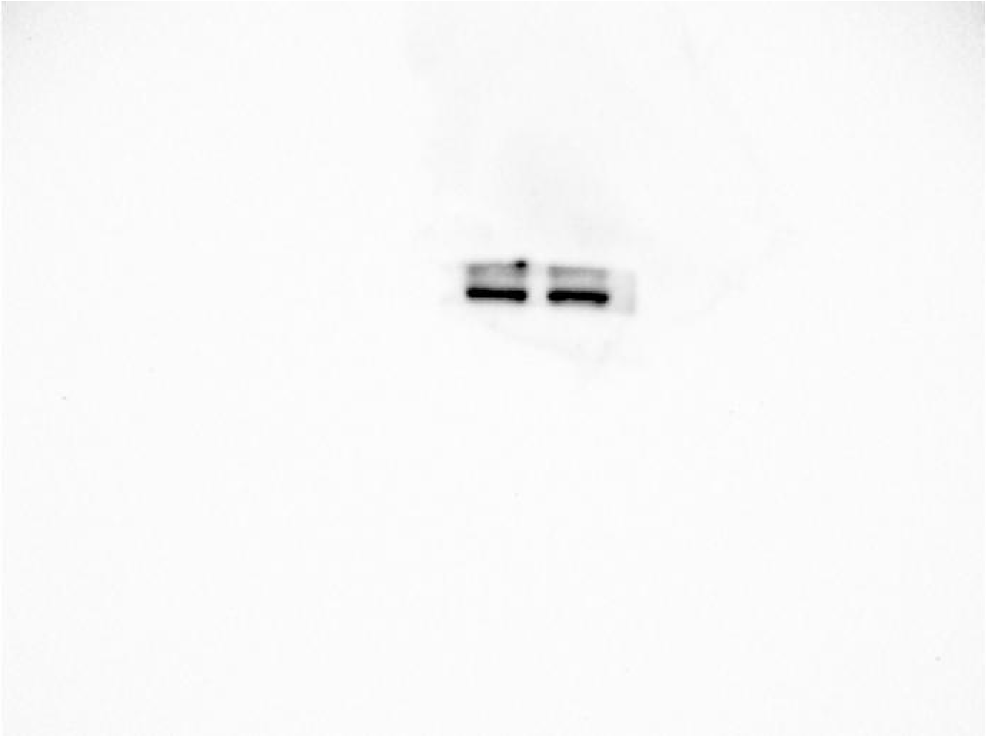

Fig. 4 A (GAPDH)

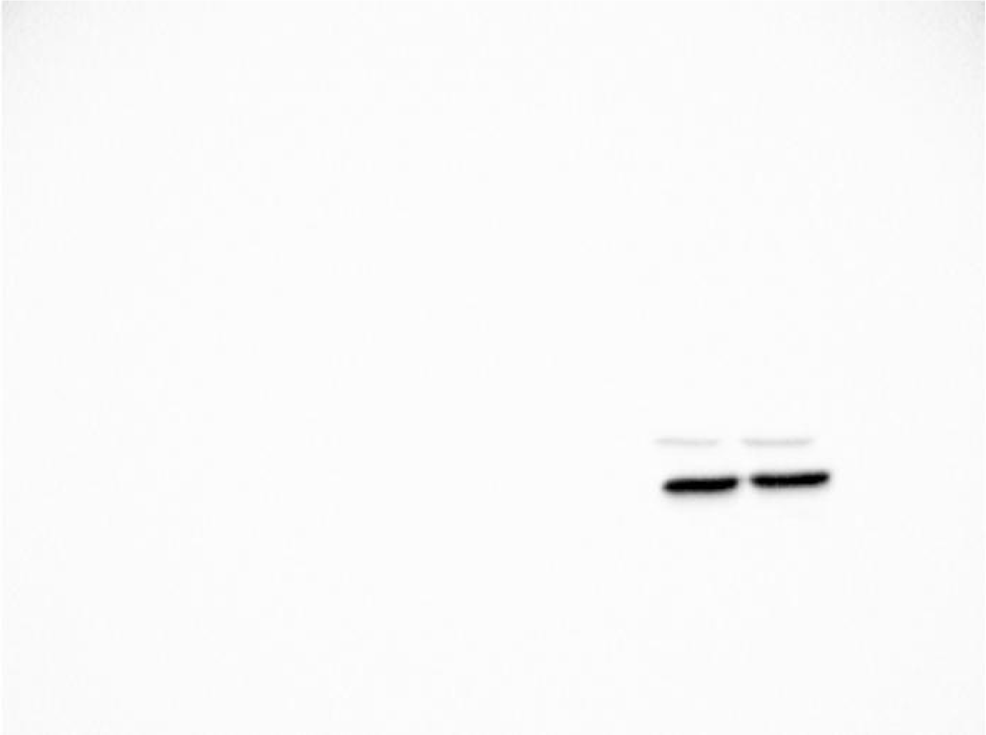

Fig. 4 B (Bcl-2)

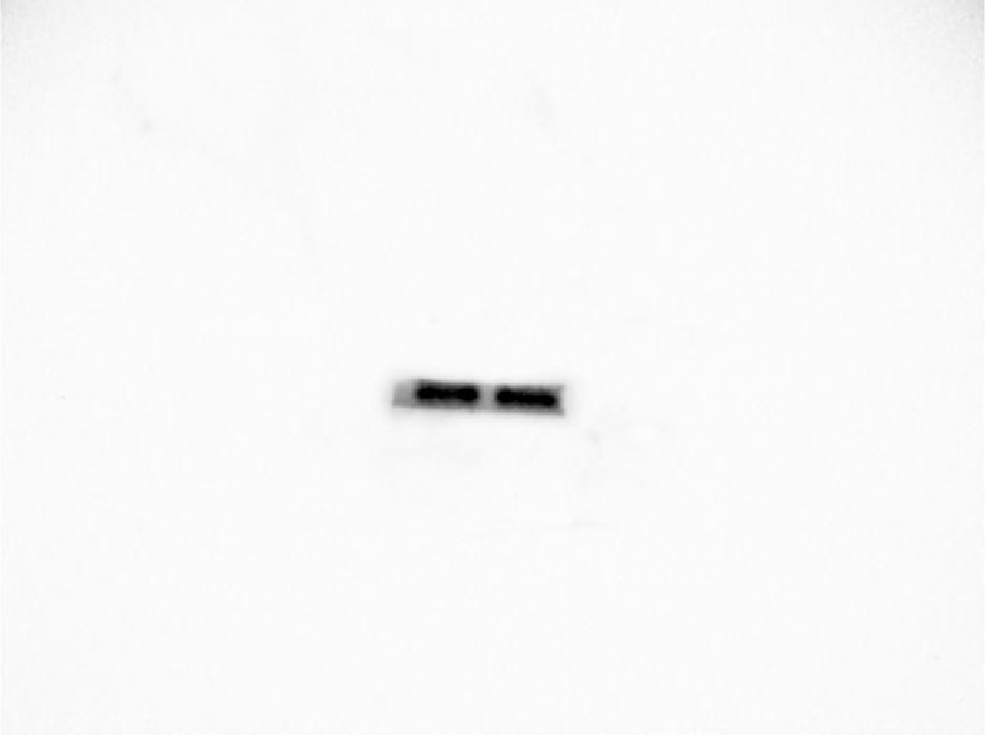

Fig. 4 B (BAX)

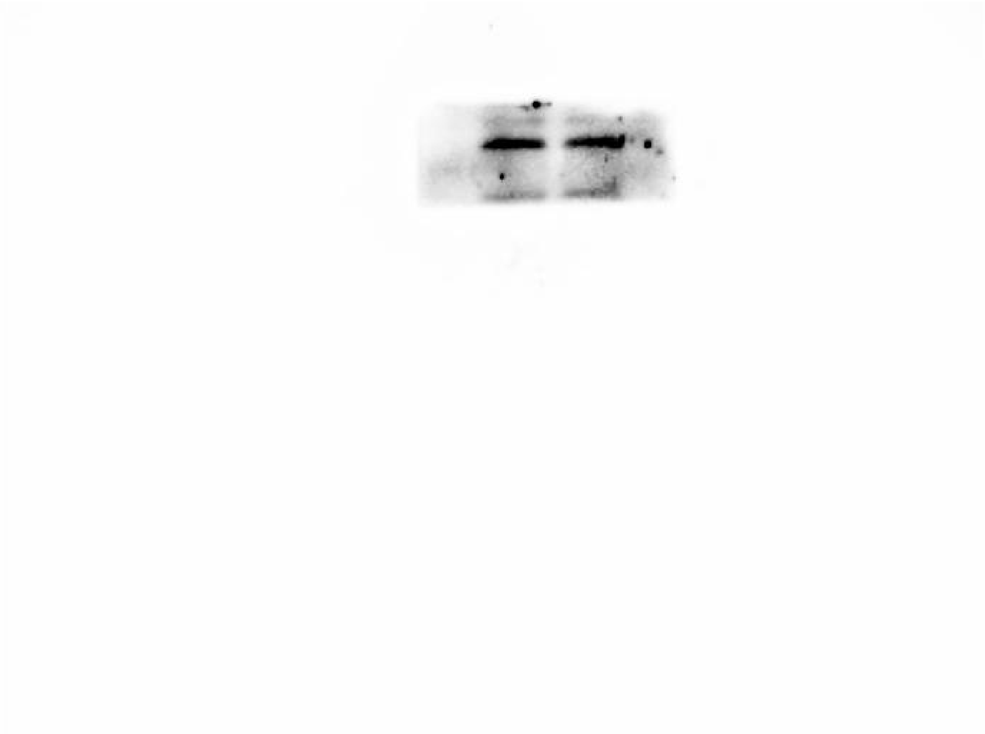

Fig. 4 B (Caspase-9)

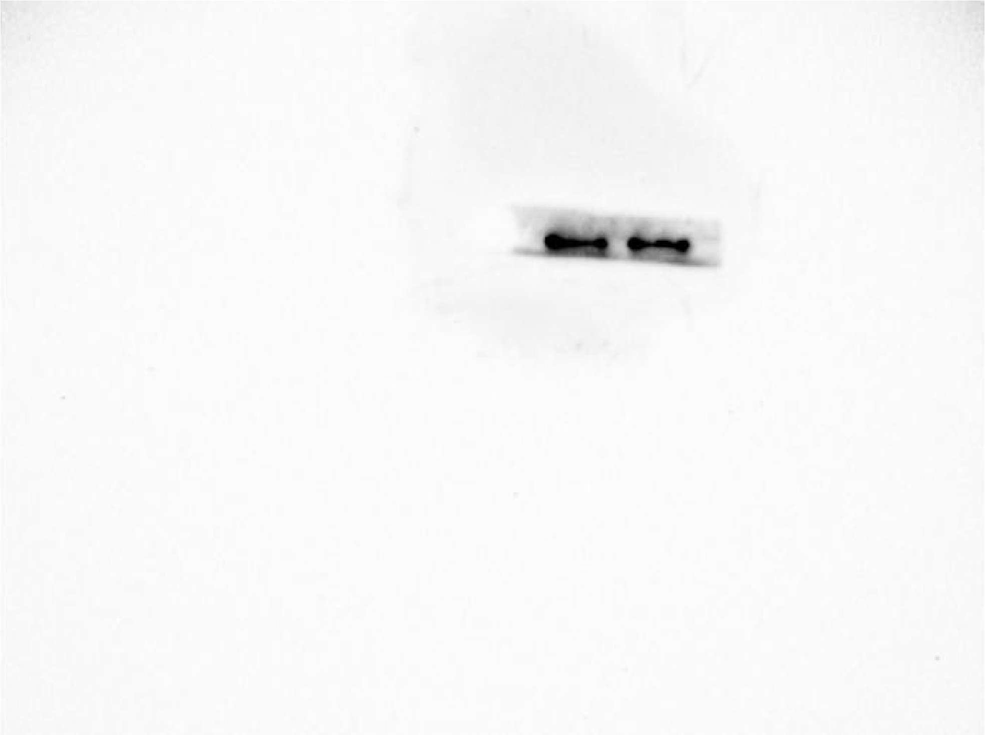

Fig. 4 B (GAPDH)

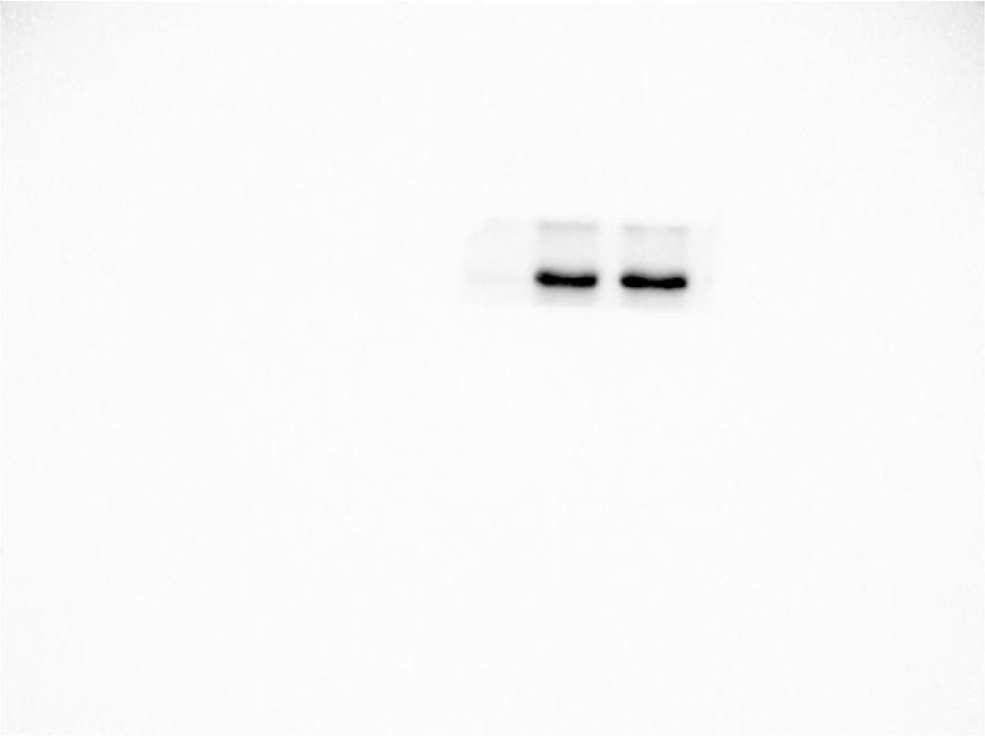

Fig. 4 E (RIP3)

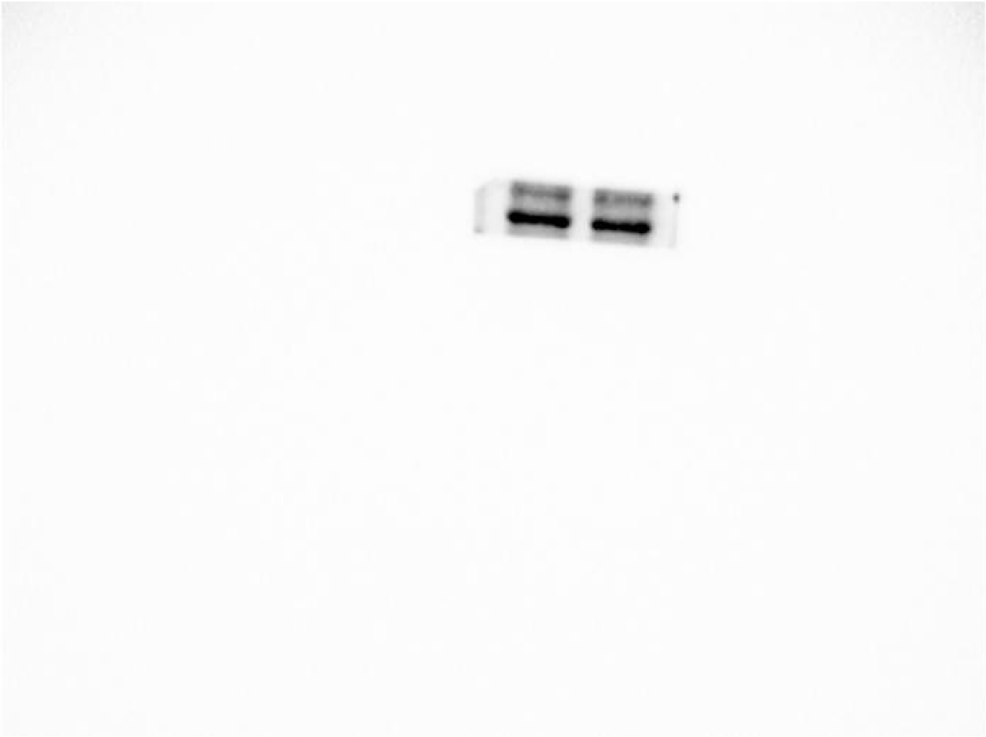

Fig. 4 E (p-RIP3)

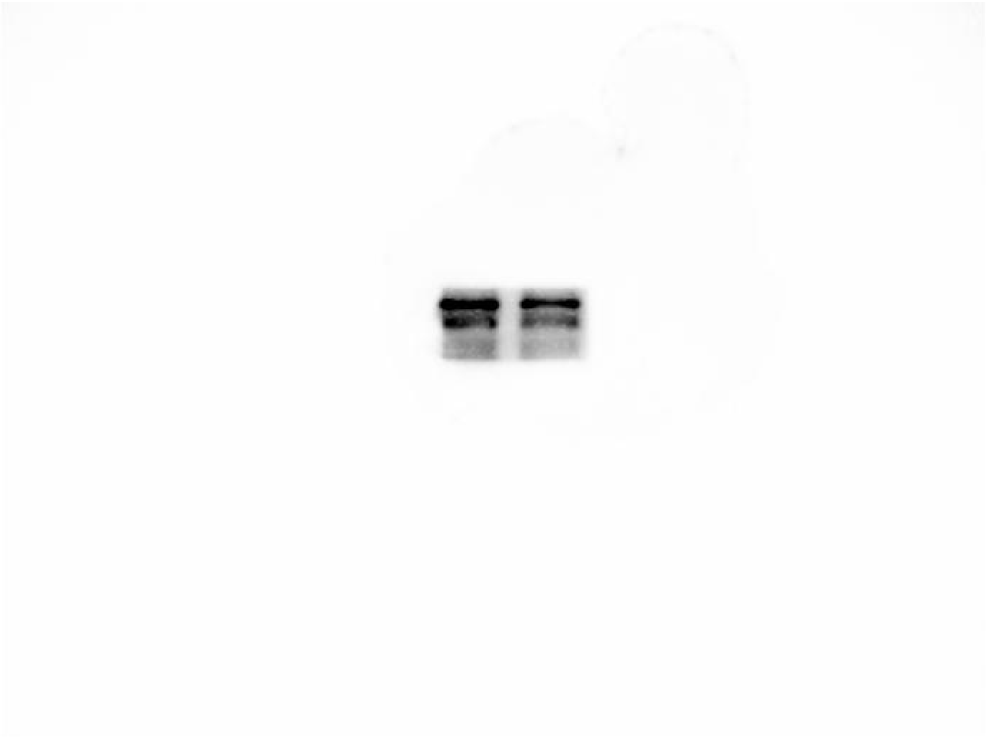

Fig. 4 E (MLKL)

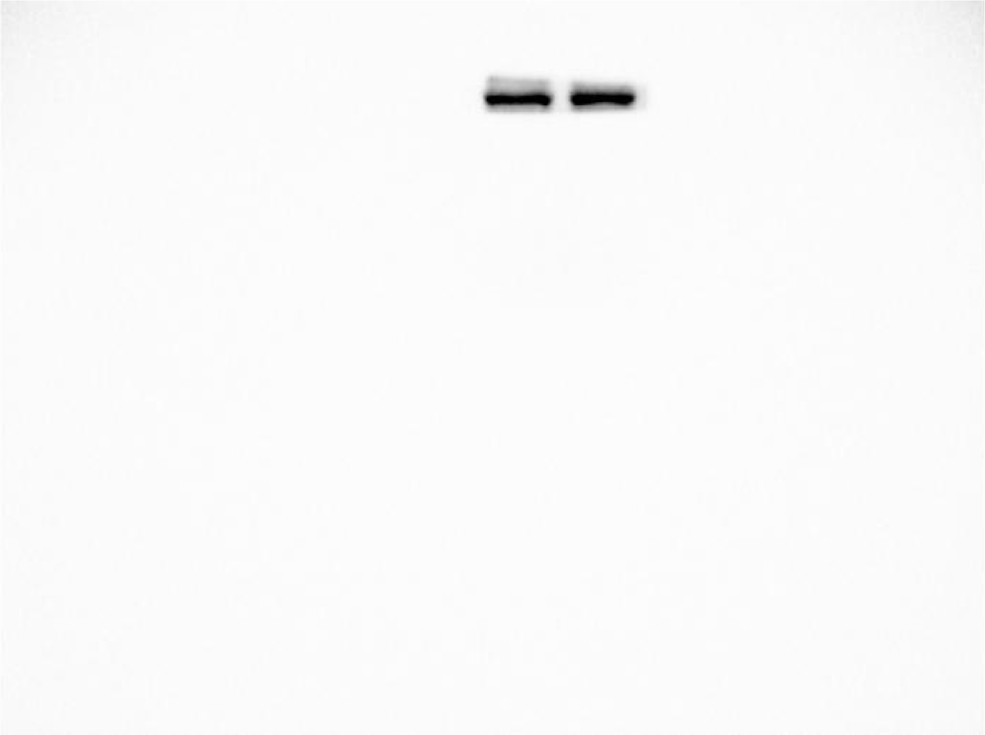

Fig. 4 E (p-MLKL)

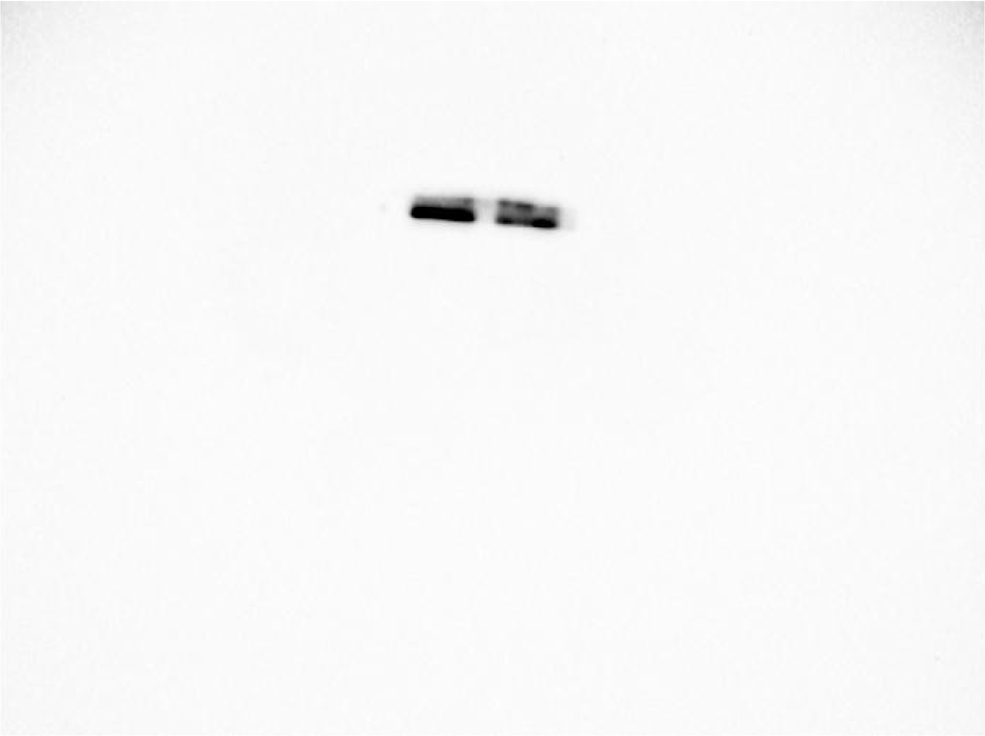

Fig. 4 E (GAPDH)

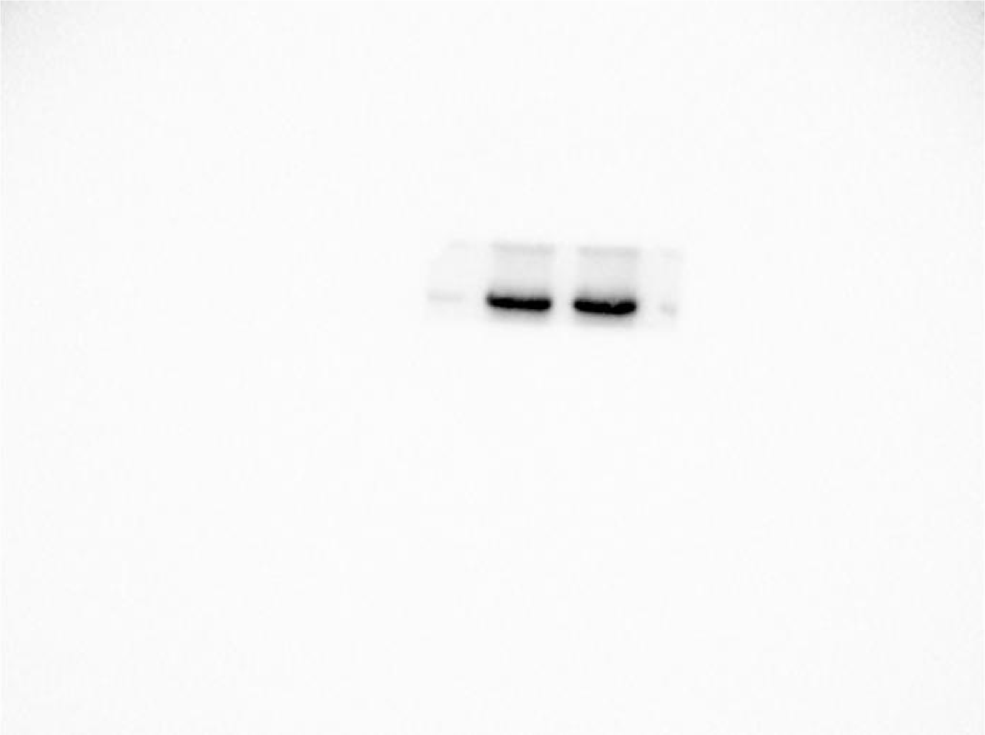

Fig. 4 F (RIP3)

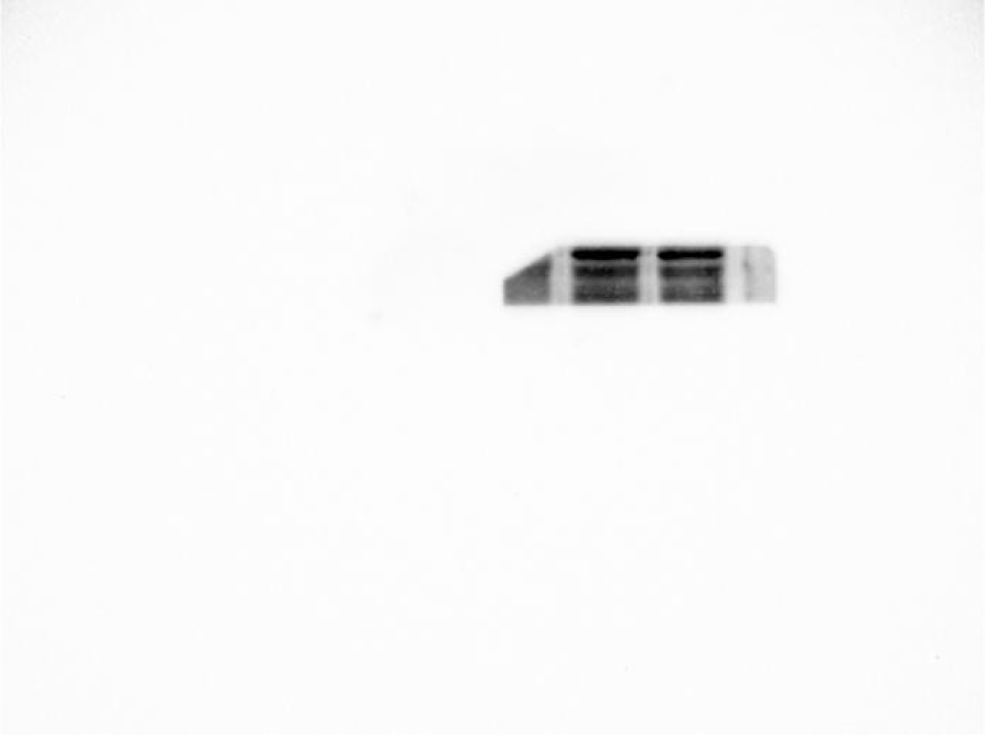

Fig. 4 F (p-RIP3)

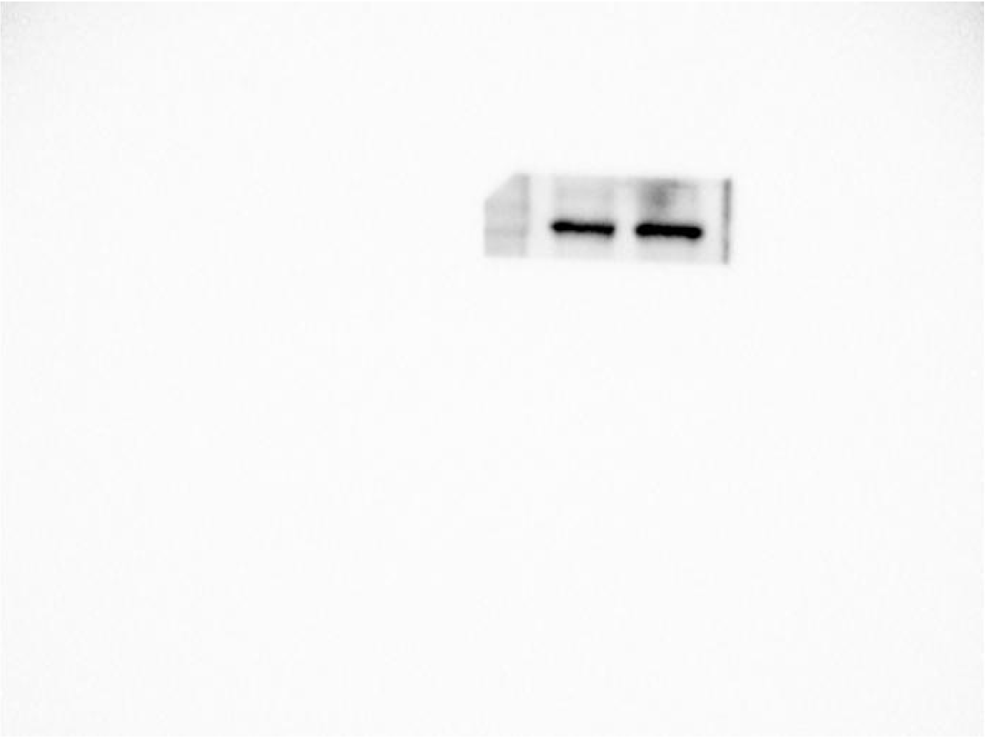

Fig. 4 F (MLKL)

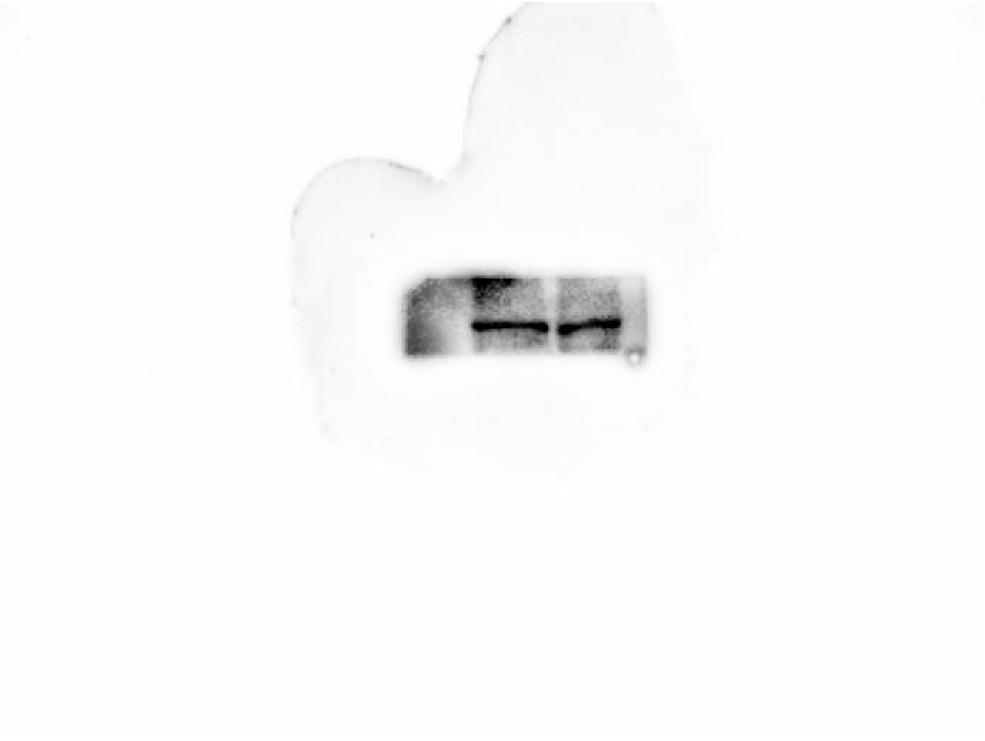

Fig. 4 F (p-MLKL)

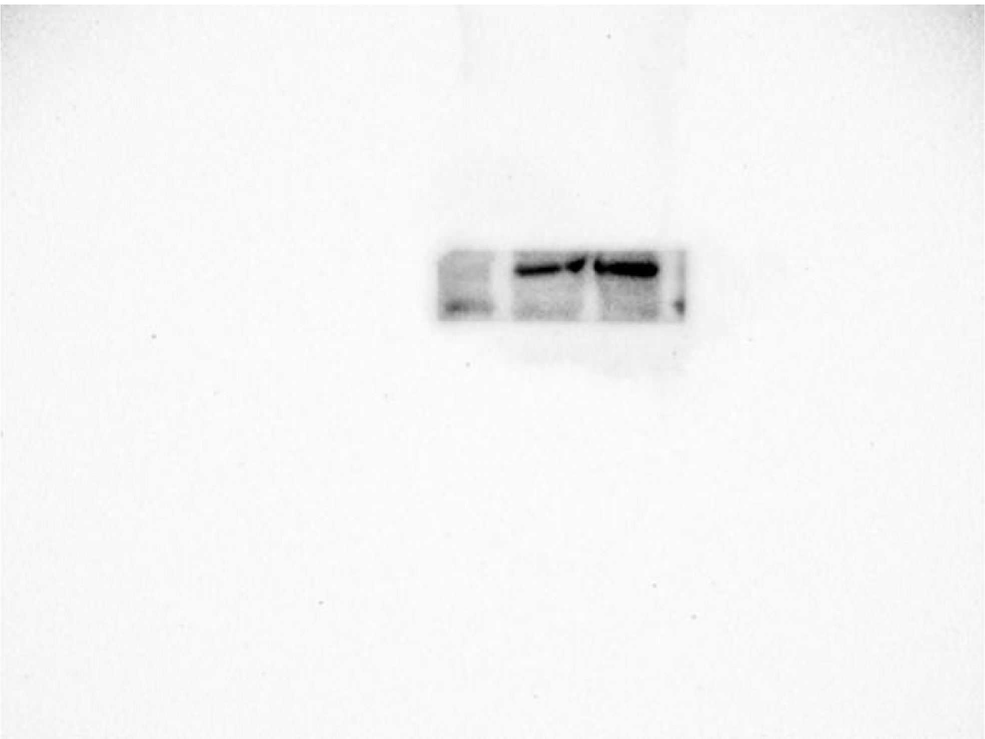

Fig. 4 F (GAPDH)

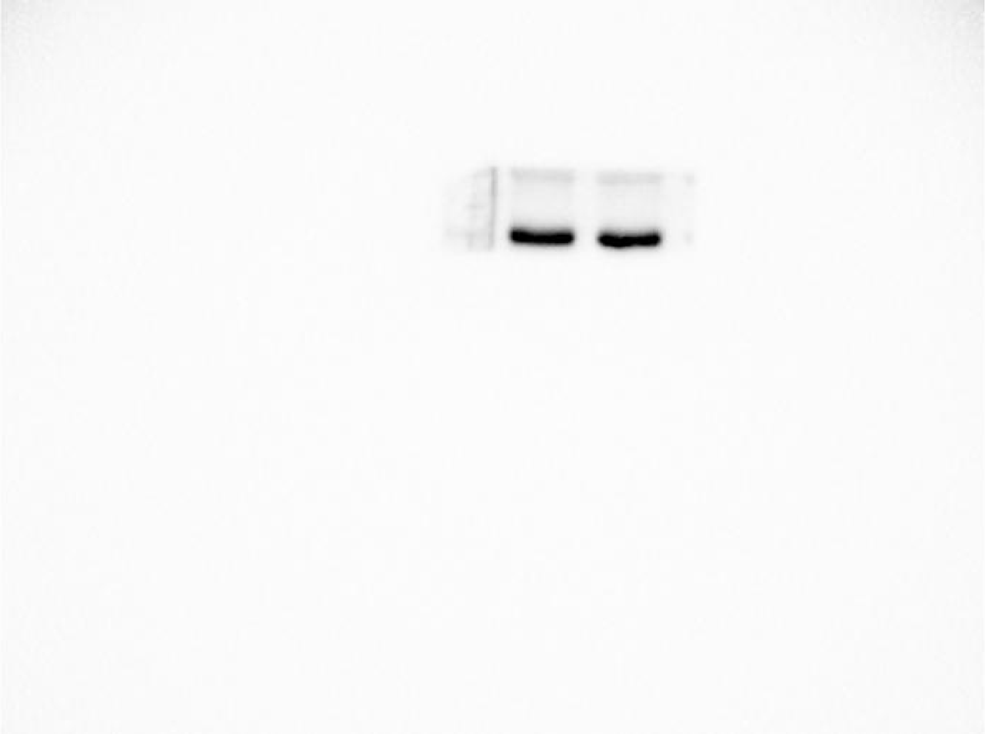

Supplement: Supplementary file 3 — Supplementary Material 3 [file 12885_2024_12308_MOESM3_ESM.pdf]
